# Evaluation of Antioxidant and Antibacterial Activities, Cytotoxicity of *Acacia seyal* Del Bark Extracts and Isolated Compounds

**DOI:** 10.3390/molecules25102392

**Published:** 2020-05-21

**Authors:** Abdirahman Elmi, Rosella Spina, Arnaud Risler, Stéphanie Philippot, Ali Mérito, Raphaël E. Duval, Fatouma Mohamed Abdoul-latif, Dominique Laurain-Mattar

**Affiliations:** 1 Université de Lorraine, CNRS, L2CM, F-54000 Nancy, France; abelfourreh@hotmail.com (A.E.); rosella.spina@univ-lorraine.fr (R.S.); arnaud.risler@univ-lorraine.fr (A.R.); stephanie.philippot@univ-lorraine.fr (S.P.); raphael.duval@univ-lorraine.fr (R.E.D.); 2 Medicinal Research Institute, Centre d’Etudes et de Recherche de Djibouti, IRM-CERD, Route de l’Aéroport, Haramous B.P. 486, Djibouti; alimerito@hotmail.fr (A.M.); fatouma_abdoulatif@yahoo.fr (F.M.A.-l.); 3 ABC Platform^®^, Faculté de Pharmacie, F-54505 Vandoeuvre-lès-Nancy, France

**Keywords:** *Acacia seyal* bark, in vitro antibacterial properties, polyphenols, antioxidant activity, tannins, MALDI-TOF

## Abstract

Water extract of *Acacia seyal* bark is used traditionally by the population in Djibouti for its anti-infectious activity. The evaluation of in vitro antibacterial, antioxidant activities and cytotoxicity as well as chemical characterization of *Acacia seyal* bark water and methanolic extracts were presented. The water extract has a toxicity against the MRC-5 cells at 256 μg/mL while the methanolic extract has a weak toxicity at the same concentration. The methanolic extract has a strong antioxidant activity with half maximal inhibitory concentration (IC_50_) of 150 ± 2.2 μg/mL using 1-diphenyl-2-picrylhydrazyl (DPPH) and IC_50_ of 27 ± 1.3 μg/mL using 2,2′-azino-bis 3-ethylbenzthiazoline-6-sulphonic acid (ABTS) radical methods. For ferric reducing/antioxidant power (FRAP) assay, the result is 45.74 ± 5.96 μg Vitamin C Equivalent (VCE)/g of dry weight (DW). The precipitation of tannins from methanol crude extract decreases the MIC from 64 µg/mL to 32 µg/mL against *Staphylococcus aureus* and *Corynebacterium urealyticum*. However, the antioxidant activity is higher before tannins precipitation than after (IC_50_ = 150 µg/mL for methanolic crude extract and 250 µg/mL after tannins precipitation determined by DPPH method). By matrix-assisted laser desorption/ionization time-of-flight mass spectrometry (MALDI-TOF MS) analysis, the results showed that the condensed tannins consist of two types of catechin and gallocatechin-based oligomers. The fractionation led to the identification of three pure compounds: two flavanols catechin and epicatechin; one triterpene as lupeol; and a mixture of three steroids and one fatty acid: campesterol, stigmasterol, clionasterol, and oleamide.

## 1. Introduction

The genus Acacia, belonging to the Fabaceae family, is composed of more than 1350 species of trees and shrubs found mostly in semi-arid places in Australia and Africa. Its fruits and bark is commonly used to tan leather or as a dye [1]. Acacia is known because of its production of Arabic gum [2] such as *Acacia nilotica*. This plant is also popular for its medicinal use and for scientific interest to search bioactive constituents. The phytochemistry of the genus Acacia differs according to the species and is not known very precisely. Several families of active chemical compounds are found in the literature such as tannins, flavonoids, alkaloids, terpenes, fatty acids, polysaccharides, anthocyanins, and saponins from different parts of Acacia [3,4,5,6]. The bark of various species of Acacia is traditionally used to treat dysentery [7], diarrhea [8], chronic pain [9], dental infection [10], and the eye’s infections [11]. The most studied species of Acacia is *Acacia nilotica.* An ethnopharmacological screening realized in Randa (Djibouti) [7] showed that different plants are emerging for their antimicrobial interest. One of these plants is *Acacia seyal*. This tree grows almost exclusively in tropical Africa. The ethanolic extracts of the bark of *A. seyal* showed in vitro antimycobacterial and cyclooxygenase inhibition [12], antibacterial [13], antimalarial [14], and anticancer activities [15]. The analysis of specialized metabolites in leaves, seeds and flowers of *A. seyal* revealed rich contents of flavonoids and saponosides [5]. However, despite the interest in the activity of this plant, to our knowledge no study has identified the chemical constituents of the bark of *A. seyal*, a traditional medicinal plant from Djibouti (Figure S1). The objective of this study was to evaluate the possible cytotoxicity, antibacterial and antioxidant activities of water, and methanolic extracts of bark of *A. seyal* as well as their chemical composition.

## 2. Results and Discussion

Before the characterization of condensed tannins and the isolation and the identification of pure compounds from *A. seyal* bark, bioactivities of crude extracts (water and methanolic extracts) were determined using total polyphenol content (TPC), total flavonoid content (TFC), extractable tannins, antioxidant activity, cytotoxicity, and antibacterial activity. The extraction yields of the bark of *A. seyal* are 13.5% using the Soxhlet apparatus with methanol as a solvent and 2.4% with water maceration.

### 2.1. Determination of Phenolic, Flavonoid Contents and Extractable Tannins

Water and methanolic extracts of *A. seyal* were evaluated for their TPC, TFC and extractable tannins and results are presented in Table 1. The total polyphenolic contents were expressed as mg gallic acid equivalent (GAE)/g of dry materials and the total flavonoid contents were expressed as mg quercetin equivalent (QE)/g of dry materials.

The amounts of total polyphenols and total flavonoids varied according to the type of bark extract studied and ranged from 1820.5 to 1927.1 mg GAE/100 g and from 13.8 and 30.9 mg QE/100 g in water and methanolic extracts respectively. Methanolic extract showed the highest polyphenolic (1927.1 mg GAE/100 g) and flavonoid (30.9 mg QE/100 g) contents. It should be noted that the flavonoid content in the methanolic extract was approximately three times higher than in the water extract, respectively 30.9 and 13.8 mg QE/100 g. Comparing these results with previous studies, it was clear that the bark of *A. seyal* contained higher flavonoid contents (up to 30.9 mg QE/100 g) than the fruits of strawberry and loquat known for their high content in flavonoids (14.6 and 14.2 mg QE/100g, respectively) [16]. To determine the tannins content, the precipitation of tannins from methanolic and water extracts was performed following the procedure described in the materials and methods section. Water and methanolic extracts of *A. seyal* bark are rich in tannins with quite similar content in both extracts (Table 1). Comparing these total results with polyphenol content, it was clear that polyphenols are mainly tannins.

It is known that condensed tannins can represent in different plant organs more than 20% of the dry material [17]. Bark of *A. confusa* showed also high levels of phenolics [18], however, bark of *A. seyal* had higher levels of extractable tannins and might be a potential source of novel natural antioxidant compounds.

### 2.2. Antioxidant Activity

The antioxidant activity using DPPH (1-diphenyl-2-picrylhydrazyl) and ABTS (2,2′-azino-bis 3-ethylbenzthiazoline-6-sulphonic acid) scavenging activities and ferric reducing/antioxidant power (FRAP) assays was investigated for: methanolic crude extract, the corresponding filtrate devoid of tannins denoted as FM and the precipitate constituted by tannins denoted as PM; water extract, the corresponding filtrate devoid of tannins denoted as FW and the precipitate constituted by tannins denoted as PW (Table 2).

Both extracts revealed a reducing power, however, the precipitate of tannins from methanolic extract showed the highest scavenging activity to DPPH radical and to ABTS radical with an IC_50_ value of 95 μg/mL and 16 μg/mL respectively. The precipitate of tannins from water extract exhibited higher DPPH and ABTS radical scavenging activities with IC_50_ values of 136 and 21 μg/mL, respectively, than the water extract, which has IC_50_ values of 183 and 33 μg/mL respectively. Furthermore, the ABTS radical scavenging ability of the methanolic and water extracts (27 and 33 μg/mL respectively) was higher to those of Vitamin C used as control (70 μg/mL). A good antioxidant activity of *A. confusa* bark extract, measured with the DPPH method, was observed with an IC_50_ of 5 μg/mL [19]. An underestimation of the antioxidant capacity was noted by the DPPH method compared to the ABTS method. This observation was also reported by Arnao [20]. This decrease would be caused by interference induced at the reading wavelength of 517 nm. The latter shows the peak absorption of DPPH unlike ABTS, which has different absorption domains. Nevertheless, this method is widely used in the evaluation of antioxidant activities. In addition, a good correlation is obtained between these two tests with *R*^2^ = 0.998 for the methanolic extract (Figure S2a). The correlation is less good for the water extract with *R*^2^ = 0.89 (Figure S2b) because of the low solubility of DPPH in water.

Thus, the use of the DPPH method may be justified but must be accompanied by other methods to conclude on the antioxidant effect of a sample. A third method of evaluating antioxidant activity was used, the FRAP assay (Table 2). In this case, the activity is expressed in equivalent amount of a positive reference, Vitamin C. The precipitate of tannins PM from methanolic extract and the methanolic extract revealed the best FRAP capacity (54.4 and 45.7 µg VCE/g respectively), while water extract, precipitate of tannins PW and filtrate FW showed weak activity (5.7, 6.7, 3.7 µg VCE/g respectively). The strong antioxidant activity of the precipitate of tannins from methanolic and water extracts may be due to the presence of condensed tannins, which are polyphenols. Indeed, the link between polyphenols and antioxidant activity is widely demonstrated in the literature [21]. Methanolic extract from leaves and fruits of *A. seyal* showed also a strong antioxidant activity (DPPH method) with IC_50_ of 76 and 77 μg/mL, respectively [22]. In this later study, also the strong antioxidant activity is linked to a high phenolic content, 10.24 and 10.1 mg GAE/g of leaves and fruits extracts respectively. In the end, PM extract and methanolic crude extract of *A. seyal* bark showed the highest antioxidant activity using DPPH and ABTS radical scavenging activities (IC_50_ with low values) and also with FRAP (high value). The precipitate and the methanolic crude extract contained phenolic compounds.

### 2.3. Cytotoxicity Activity

**Figure 1:**
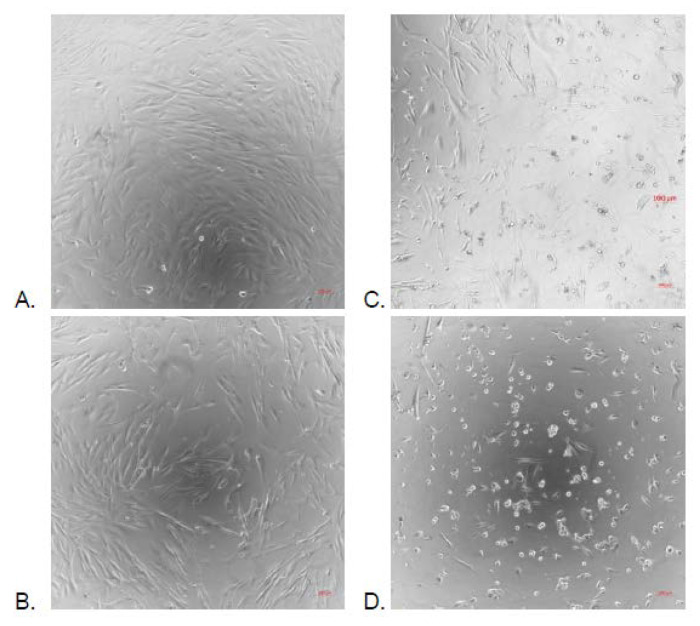
Microscopic observation of the effect of water *Acacia seyal* extract at different concentrations on MRC-5 cells at 24 hours. (**A**): DMSO control, (**B**): 64 μg/mL, (**C**): 128 μg/mL, (**D**): 256 μg/mL, observation under ×10 air objective.

Methanolic crude extract and water extract were evaluated for their cytotoxicity at various concentrations (100 μg/mL, 128 μg/mL and 256 μg/mL) against embryonic lung MRC-5 cell line using MTT test. Methanolic extract at 100 μg/mL and 128 μg/mL did not show any cytotoxic activity against MRC-5 cells (100% and 98% of cell survival respectively), while they showed moderate toxicity at a concentration of 256 μg/mL (78.17% of cell survival). However, the water extract exhibited an increasing toxicity against MRC-5 cells depending on the increasing concentrations tested. This toxicity was shown by the decrease of the cell density and modification of the morphology with microscopic observations (Figure 1). The picture of culture in cell layers could correctly report both the decrease in the number of cells and the change in the morphology of the survivors. The viability could not have been measured since water extract alone was able to convert tetrazolium salt in formazan.

Additional technic of analysis with neutral red was also performed but failed to produce reliable measurement. For more investigations, the precipitate of tannins from water extract PW and the fraction devoid of tannin from water extract FW were evaluated for their toxicity and a concentration causing 50% of cell death is sought. Precipitate of tannins PW has an IC_50_ of 241.2 ± 16.8 μg/mL while the fraction devoid of tannin FW is not toxic because the cell viability at 1024 μg/mL that represented the highest concentration tested is 88.9 ± 5.2%. Tannins could therefore be responsible for the cellular toxicity of the water extract observed. However, methanolic extract containing the same percentage of tannins as water extract (82.8% and 80.7% respectively) showed no toxicity against MRC-5 cell line. It has been reported that phenolic compounds showed the growth inhibition in breast cancer cells [22] and they have been suggested as responsible for the cytotoxic effects of *Baccharis* species [23] whereas flavonoids induced cytotoxicity in various human cell lines [24]. Both flavonoid and total polyphenolic compound contents are higher in non-toxic methanolic extract than in toxic water extract of *A. seyal*. These results suggest that it could be quality other than quantity that matters on the potency of phenolic compounds. The pure compound ƴ-sitosterol isolated from *Acacia nilotica* leaves showed anticancer properties [25]. This compound was also isolated from the no toxic methanolic extract of *A. seyal*. This suggests that the cytotoxicity observed in water extract of *A. seyal* bark could be from other specialized metabolites apart from this compound and phenolics.

### 2.4. Antibacterial Activity

The crude water and methanolic extracts were evaluated for the antibacterial activities against nine human bacterial pathogen strains: *Escherichia coli*, *Staphylococcus aureus*, *Pseudomonas aeruginosa*, *Corynebacterium urealyticum*, *Shigella sonnei*, *Salmonella enteric sv. typhi*, *Klebsiella pneumoniae*, *Staphylococcus epidermidis,* and *Streptococcus agalactiae*. Water extract was devoid of any antibacterial activity against all bacterial strains tested except against *Staphylococcus aureus* with a MIC value of 512 µg/mL. The methanolic extract showed a significant activity against two Gram-positive bacteria: *Staphylococcus aureus* and *Corynebacterium urealyticum* with MIC values of 64 µg/mL and one Gram-negative bacteria: *Pseudomonas aeruginosa* with a MIC value of 512 µg/mL. The antibacterial activity of methanolic extracts devoid of tannins (FM) and free tannin precipitate from methanolic extracts (PM) was evaluated, and results of the MIC values are presented in Table 3.

The methanolic fraction devoid of tannins FM showed significant activity against *S. aureus* and *C. urealyticum* (MIC values of 32 µg/mL) and against *P. aeruginosa* (MIC value of 512 µg/mL) while the crude methanolic extract showed a lower activity against *S. aureus* and *C. urealyticum* (MIC values of 64 µg/mL). Surprisingly, the improvement in potency of the fraction FM devoid of tannins seemed to indicate that the tannins were antagonists to the antibacterial activity. Tannins are water-soluble and the results showed that the MIC values of both water extract and precipitate of tannins from water extract PM were similar against the tested strains. However, tannins are known to possess antibacterial activity by their capacity to form bonds with glycoproteins present on the external membrane of bacteria [26]. This leads to suggest that the antibacterial activity observed with the fraction devoid of tannin FM was due to other substances. Also extracts of *Bolusanthus speciosus* with small amount of phenolics and no tannin detected showed the highest antimicrobial effects [27]. The synergistic antibacterial effects of polyphenolic compounds have been previously reported in the literature [28]. The sensitivity of *S. aureus*, *P. aeruginosa* and *C. urealyticum* to the methanolic extract devoid of tannins FM and its 12 fractions (F1 to F12) as well as isolated compounds **1**, **2** and **3** was carried out (Table 3). Reduction in potency of the methanolic extract devoid of tannins FM when fractionated was observed except for the fraction F12, which showed the same significant activity against *S. aureus* as FM (MIC value of 32 µg/mL) and the fractions F5, F8 and F11, which showed higher activity against *S. aeruginosa* (128 µg/mL) than the extract FM (>256). Fractions F2, F5, F7, and F8 possessed activity against *S. aureus* with an MIC value of 128 μg/mL. Against *C. urealyticum*, only the fractions F5 and F12 showed an activity (MIC value of 128 µg/mL) that was lower than the extract FM (MIC value of 32 µg/mL). The pure compounds **1**, **2** and **3** did not show any antibacterial activity.

### 2.5. Chemical Constituents

The fractions F4, F5 and F12 were further investigated for their phytochemical characteristics. One triterpene was identified from F4 by GC-MS and ^1^H-NMR as lupeol (compound **1**). Two flavanols were also isolated from F12 and their structure was determined by NMR and mass fragmentation. The structures are confirmed by comparison with the literature data [29] and authentic specimens as epicatechin (compound **2**) and catechin (compound **3**). The original spectra are shown in the Supplementary Material.

**Figure 2:**
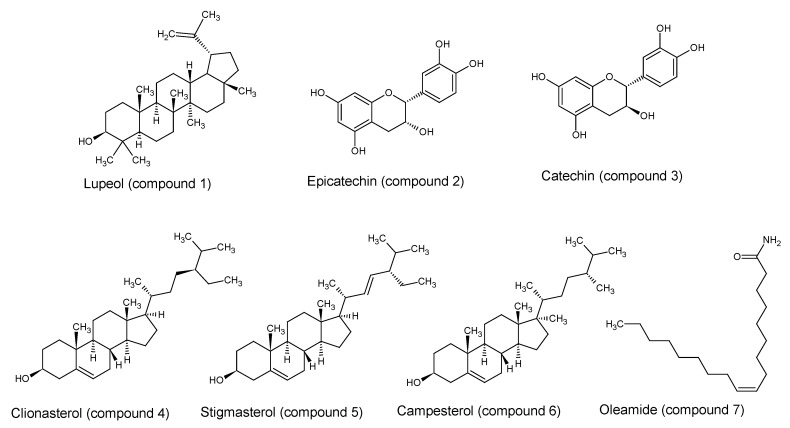
Identified compounds in *A. seyal* bark.

Four compounds were identified in the fraction F5 by GC-MS; three steroids as clionasterol (compound **4**), stigmasterol (compound **5**), and campesterol (compound **6**); and one fatty acid as oleamide (compound **7**) [30]. All structures are presented in Figure 2.

The description of the isolated compounds (**1**, **2**, **3**) and the compounds identified by GC-MS (**4**, **5**, **6**, **7**) is: Compound **1**, Lupeol: (C30H50O, RetIndex: 2848), GC-MS: *m/z* = 426 (38) [M^+^], 411 (7), 316 (11), 257 (10), 207 (71), 189 (82), 135 (66), 81 (100), 55 (61). ^1^H-NMR (400 MHz, CDCl_3_): *δ* (ppm) 4.62 (d, 1H, *J* = 2.45), 4.50 (dd, 1H, *J* = 2.45, 1.32), 3.11 (dd, 1H, *J* = 11, 5.3), 2.30 (td, 1H, *J* = 11, 5.8), 1.86–1.79 (m), 1.61 (s, CH3), 1.46–1.38 (m), 1.27–1.21 (m), 1.19 (s, CH3), 1.13–1.00 (m), 0.95 (s, CH3), 0.89 (s, CH3), 0.83–0.77 (m), 0.75 (s, CH3), 0.72 (s, CH3), 0.69 (s, CH3). Compound **2**, Epicatechin was obtained as brown powder. ^1^H-NMR (400 MHz, CD_3_OD): *δ* (ppm) 6.98 (d, 1H, H-2′, *J* = 1.2), 6.8 (dd, 1H, H-6′, *J* = 8.2, 1.5), 6.76 (d, 1H, H-5′, *J* = 8.2), 5.96 ppm (d, 1H, H-6, *J* = 2.2), 5.94 ppm (d,1H, H-8, *J* = 2.3), 4.82 (sbr, 1H, H-2), 4.17 (m, 1H, H-3), 2.86 (d, 1H, H-4a, *J* = 16.6, 4.7), 2.74 (dd, 1H, H-4b, *J* = 16.5, 2.7). ^13^C-NMR (100 MHz, CD_3_OD): *δ* (ppm)158.1 (C-7), 157.7 (C-5), 157.4 (C-9), 146.0 (C-4′), 145.8 (C-3′), 132.4 (C-1′), 119.5 (C-2′), 116.0 (C-5′), 115.5 (C-6′), 100.2 (C-10), 96.5 (C-6), 96.0 (C-8), 80.0 (C-2), 67.6 (C-3), 29.3 (C-4). HRESIMS (negative mode) *m/z* 289.0726 [M-H]^−^ (calcd. for C_15_H_13_O_6_, 289.0712). Compound **3**, Catechin was obtained as brown powder. ^1^H-NMR (400 MHz, CD_3_OD): *δ* (ppm); 6.85 (d, 1H, H-6′, *J* = 2,0), 6.76 (d, 1H, H-5′, *J* = 8.0), 6.72 (dd, 1H, H-2′, *J* = 8.2, 2.0), 5.93 (d, 1H, H-6, *J* = 2.2), 5.86 (d, H-8, *J* = 2.3), 4.57 (d, 1H, H-2, *J* = 7.6), 3.97 (td, 1H, H-3, *J* = 7.8, 5.6), 2.86 (dd, 1H, H-4a, *J* = 16.1, 5.4), 2.49 (dd, 1H, H-4b, *J* = 16.1, 8.1). ^13^C-NMR (100 MHz, CD_3_OD): *δ* (ppm) 157.9 (C-7), 157.7 (C-5), 157.0 (C-9), 146.31 (C-4′), 146.33 (C-3′), 132.4 (C-1′), 120.1 (C-2′), 116.2 (C-5′), 115.4 (C-6′), 100.9 (C-10), 96.4 (C-6), 95.6 (C-8), 82.9 (C-2), 68.9 (C-3), 28.3 (C-4). HRESIMS (negative mode) *m/z* 289.0719 [M-H]^−^ (calcd. for C_15_H_13_O_6_, 289.0712). Compound **4**, Clionasterol (C29H50O, RetIndex: 2731), GC-MS: *m/z* = 414 (100) [M^+^], 354 (5), 329 (42), 255 (30), 213 (31), 145 (44), 81 (66), 43 (87). Compound **5**, Stigmasterol (C29H48O, RetIndex: 2739), GC-MS: *m/z* = 412 (59) [M^+^], 351 (59), 300 (27), 255 (45), 213 (13), 145 (39), 83 (100), 55 (90), 43 (33). Compound **6**, Campesterol (C28H48O, RetIndex: 2632), GC-MS: *m/z* = 400 (100) [M^+^], 367 (23), 315 (55), 255 (27), 213 (30), 159 (43), 145 (50), 81 (61), 43 (89). Compound **7**, Oleamide (C18H35NO, RetIndex: 2228), GC-MS: *m/z* = 281 (7) [M^+^], 126 (8), 72 (86), 59 (100), 55 (36).

### 2.6. Characterization of Condensed Tannins by MALDI-TOF MS Analysis

**Figure 3:**
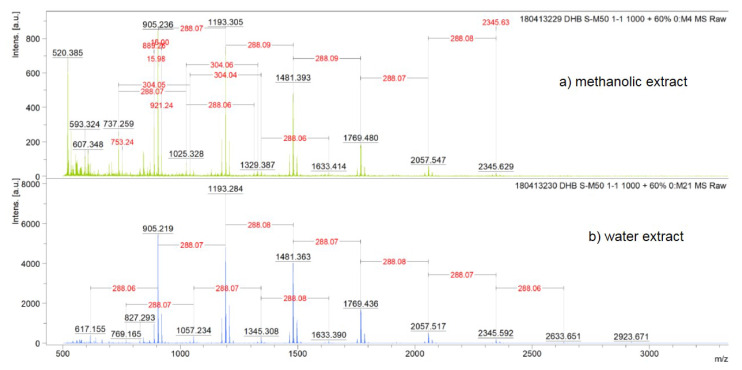
MALDI-TOF positive reflectron mode mass spectra of the condensed tannins from methanolic (**a**) and water (**b**) extracts of *Acacia seyal* bark.

The structure of tannin oligomers isolated from methanolic and water extracts of *A. seyal* bark as well as their degree of polymerization were investigated by matrix-assisted laser desorption/ ionization time-of-flight (MALDI-TOF) mass spectrometry. It is a suitable method for examining tannin oligomers [31]. Figure 3 shows the MALDI-TOF mass spectra of the polymeric tannin mixtures of both extracts, recorded as Na^+^ adducts in the positive ion reflectron mode.

The theoretical molecular weight of each peak is calculated, using equation (1), from combinations of its repeating units [32]:

(1) M+Na=23 (Na)+2 (endgroups, 2×H)+304×N1+288×N2+152×N3

The polymeric character is reflected by the periodic peak series representing different polymers. Condensed tannins isolated from the methanolic and water extracts are characterized by mass spectra with a series of peaks with various molecular weights from 737.3 to 2345.6 Da and from 617 to 2345.6 Da respectively. Two types of sequences were identified for the condensed tannins of the methanolic extract (S1 and S2) and of the aqueous extract (S3 and S4) (Table 4).

**Figure 4:**
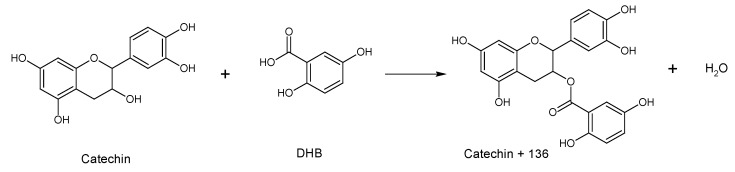
Esterification of catechin with the matrix molecule (DHB: 2,5 dihydroxybenzoic) during the MALDI-TOF experiment.

In the S1 series of the methanolic extract, the peaks correspond to combinations of these two monomers, epi/catechin (distance of 288 Da) and gallo-epi/catechin (distance of 304 Da), added by 136 Da that correspond to the product of an esterification between the catechin and the matrix, which is a carboxylic acid. This esterification could take place during the MALDI-TOF analysis, between the catechin and the DBH (2,5 dihydroxybenzoic) matrix. This produces a catechin monomer increased by 136 Da to the molar weight, a phenomenon already reported by Duval et al. [33] (Figure 4).

## 3. Materials and Methods

### 3.1. Chemicals

1,1-Diphenyl-2-picrylhydrazyl (DPPH), 2,2′-azinobis-3-ethylbenzothiazoline-6-sulfonic acid (ABTS), Vitamin C, Trolox, iron (II) sulfate (FeSO_4_), iron (III) chloride (FeCl_3_), Folin–Ciocalteu reagent (FC reagent), hydrochloric acid (HCl), sulphuric acid (H_2_SO_4_), organics solvants, trichloroacetic acid (TCA), dimethyl sulfoxide (DMSO), potassium persulfate, sodium dihydrogen phosphate, sodium hydroxide (NaOH), CD_3_OD and CDCl_3_ were purchased from Sigma Aldrich (St Quentin Fallavier, Franceor Acros organics, part of Thermo Fischer (Illkirch, France). The solvents used are HPLC profile grade.

### 3.2. Plant Materials

The bark of *Acacia seyal* Del. (Fabaceae) was collected in the locality of Day from the Tadjourah region, North of Djibouti in December 2015 (Latitude: 11°48’16.8660” N, Longitude: 42°37’46.1256” E). The identification was carried out by Prof. Maha Kordofani of the University of Khartoum of Sudan and its floor filed under No. 46/HND/2015 to the national herb of Djibouti.

### 3.3. Preparation of Plant Extracts

First the bark of *A. seyal* was crushed. Six-hundred-and-fifteen grams of the dry powder was extracted with 5 liters of methanol in Soxhlet apparatus for 7 hours and then evaporated under reduced pressure to dryness to obtain 82.3 g. Two-hundred grams of the dry powder was extracted with 600 mL of water using maceration during 30 h as indicated in the traditional method and then lyophilized leading to 4.9 g.

### 3.4. Precipitation of Tannins from Crude Extracts

Tannins present in methanolic and water extracts were precipitated according to the protocol indicated by Lhuillier [34] with some light modifications to replace chloroform by dichloromethane. To 3 g of both solid crude extracts were added 80 mL of methanol as solvent in two separated flasks. Then dichloromethane was added in a ratio of 8:92 (methanol: dichloromethane, *v/v*). Spontaneously, the tannins precipitated. The mixture thus obtained was left for 3 hours at 4 °C and then filtered. After evaporation, methanolic extract devoid of tannins was noted as FM and the precipitate of tannins from methanolic extract was noted as PM. From water extract, the filtrate noted as FW corresponds to the water extract devoid of tannins and PW to the tannins precipitate.

### 3.5. Determination of the Total Phenol Content (TPC) in the Extracts

The TPC of all extracts was evaluated using the Folin–Ciocalteu method [35] with some modification. Briefly, 100 mL of the extract solution (10 mgmL^−^1 MeOH) was shaken for 3 min with 1 mL of diluted (1:10) Folin–Ciocalteu reagent. Then, 900 mL of 10% Na_2_CO_3_ was added and the final volume was made up to 5.0 mL with distilled water. After the mixture was allowed to stand for 2 h at room temperature, the absorbance at 725 nm was measured using a UV-vis spectrophotometer.

The results of TPC were estimated using a standard curve prepared using gallic acid and expressed as milligram GAEs per gram of extract on dry weight basis.

### 3.6. Determination of the Total Flavonoid Content (TFC) in the Extracts

The total flavonoid content was determined using [36] method as adapted by [37]. Briefly, sample solution (1 mg/mL; 1 mL) was mixed with the same volume of aluminium trichloride (2%) in methanol. Similarly, a blank was prepared by adding sample solution (1 mL) to methanol (1 mL) without AlCl_3_. The sample and control absorbance were read at 415 nm after a 10 min incubation at room temperature. The absorbance of the blank was subtracted from that of the sample. Quercetin was used as a reference standard and the total flavonoid content was expressed as milligrams of quercetin equivalents (mg QE/g extract).

### 3.7. Preparation of Antioxidant Activities Tests

#### 3.7.1. DPPH Method

The free-radical-scavenging activity of the extracts of *A. seyal* was measured using an improved DPPH assay [38]. The extract solution with concentration of 0.3 mL was mixed with a solution of 0.2 mmol/L DPPH in methanol (2.7 mL). The mixture was mixed vigorously and then left to stand for 1 h at room temperature before measuring the absorbance value at 517 nm. The percent of inhibition rate of radical scavenging activity was calculated using the following Equation (2):

(2) % Inhibition =[(As−Ai)/As]×100

where “As” is the absorbance of DPPH alone, and “Ai” is the absorbance of DPPH in the presence of various extracts. The concentrations of Trolox and Vitamin C (VC) identical to the experimental samples were used as reference.

#### 3.7.2. ABTS Method

The ability of the extract to scavenge ABTS radical was determined according to a previously published method [39]. ABTS was dissolved in deionized water at 7 mmol/L concentration, and potassium persulfate with a concentration of 2.45 mmol/L was added afterward. The reaction mixture was kept in the dark at room temperature for 16 h. The mixture was then diluted with 80% ethanol to obtain an absorbance value of 0.700 ± 0.005 at 734 nm. Test substances (0.3 mL) at various concentrations were incubated with ABTS + solution (2.7 mL) in a 30 1C water bath for 30 min in the dark. The absorbance at 734 nm was immediately recorded. Samples of BHT and VC at the same concentrations were used as references. The level of radical scavenging was calculated using the aforementioned equation for DPPH.

#### 3.7.3. FRAP Method

The FRAP assay was carried out according to [40] with slight modification. Briefly, the FRAP reagent was prepared from acetate buffer (pH 3.6), 10 mmol TPTZ solution in 40 mmol HCl and 20 mmol iron (III) chloride solution in proportions of 10:1:1 (*v/v*), respectively. The FRAP reagent was prepared fresh daily and was warmed to 37 C in a water bath prior to use. Fifty microliters of sample were added to 1.5 ml of the FRAP reagent. The absorbance of the reaction mixture was then recorded at 593 nm after 4 min. The standard curve was constructed using Vitamin C solution (0.065–33.3 µg/ml), and the results were expressed as µmol VCE/g dry weight of plant material. All the measurements were taken in triplicate and the mean values were calculated.

### 3.8. Cytotoxicity Assay

The toxicity of the extracts on cell viability was evaluated on MRC-5 cells, a fibroblast derived from normal lung tissue (ECACC, reference 05090501) using the MTT test, based on the reduction of MTT in formazan crystals by metabolically active cells [41]. MRC-5 cells is a line of normal cells (non-cancerous) which have the capacity to multiply under in vivo culture conditions. This cell line is commonly used as a model for the evaluation of toxicity. Cells were plated at 10000 cells per well in complete medium in 96-well tissue culture plates and grown for 48 hours at 37 °C. Then medium was discarded and replaced by fresh medium containing extracts previously dissolved in dimethyl sulfoxide. Three different controls were added: medium alone, cells in medium and extract in medium. After 24 h incubation, medium was replaced by 100 µl of 0.5 mg MTT solution in medium per well and plates were incubated for 4 hours at 37 °C. Then formazan crystals were dissolved by 100 µl of dimethyl sulfoxide. Absorbance was measured at 540 nm using a 96-well plate reader. Percentages of survival and half maximal inhibitory concentration (IC_50_) were calculated using MS Office Excel. The water extract was able to convert MTT in formazan. That is why a confirmation of its toxicity was carried out by microscopic observation.

### 3.9. Antibacterial Assay

For experiments, the following bacteria have been used:

*Escherichia coli* ABC5 (ATCC 25922), *Shigella sonnei* ABC16 (ATCC 9290), *Salmonella enterica* sv. *typhi* ABC17 (ATCC 13311), *Staphylococcus aureus* ABC1 (ATCC 29213), *Pseudomonas aeruginosa* ABC4 (ATCC 27853), *Klebsiella pneumoniae* ABC12 (ATCC 700603), *Streptococcus agalactiae* ABC6 (ATCC 27956), *Staphylococcus epidermidis* ABC91 (clinical origin), and *Corynebacterium urealyticum* ABC145 (clinical origin).

To evaluate the antibacterial activities of the compounds, the Minimal Inhibitory Concentration (MIC) was determined by broth microdilution method based on ISO 20776-1:2006 standard [42], in accordance with CLSI [43] and EUCAST [44] guidelines.

Bacterial suspensions were prepared by suspending three to five isolated colonies from Mueller-Hinton Agar, Cation-Adjusted (MHA-CA) in 5 mL of Mueller-Hinton Broth, Cation-Adjusted (MHB-CA).

After 24 h of growth at 35 °C, the medium was removed by centrifugation (5 min at 3000 g) and cells were resuspended in 0.85% NaCl to obtain a 0.5 McFarland suspension.

This suspension was then adjusted in MHB-CA at 1 × 10^6^ [0.4 × 10^6^ − 1.6 × 10^6^] CFU/mL.

Twofold serial dilutions of the compounds (prediluted in DMSO) were prepared in 0.05 mL MHB-CA in round-bottom, untreated 96-well plates. An equal volume (0.05 mL) of bacterial inoculum was added to each well. The final concentration of DMSO never exceeded 5% of the final volume. Each condition was repeated in the eight lines of the microplate.

Final bacterial concentration in the wells was 5 × 10^5^ [2 × 10^5^ − 8 × 10^5^] CFU/mL and checked as described in ISO 20776-1:2006 standard.

In each line, a positive growth control (compound diluent, without compound) and two negative controls (0.05 mL MHB-CA with or without compound at the highest assayed concentration, and without bacteria) were included.

After incubation for 18h at 35 °C, the MICs were visually determined as recommended by ISO standard. In all experiments, bacteria were tested against vancomycin (for Gram positive bacteria) and gentamicin (for Gram negative bacteria) by microdilution method according to ISO 20776-1:2006 as control. Expected results were obtained (MIC ≤ 1 mg/L).

### 3.10. Isolation and Characterization of Pure Compounds

#### 3.10.1. Isolation of Pure Compounds

The methanolic extract devoid of tannins FM was subjected to flash chromatography eluted with toluene: ethyl acetate (75:15 *v/v*). A total of 12 fractions were obtained (F1–F12) on combining the eluates according to their similarity behaviour on TLC. Fraction F4 (45 mg) was submitted to preparative TLC using the previous mobile phase. One pure compound was obtained from F4 (2 mg, compound **1**). Fraction F12 (2032 mg) was subjected to flash chromatography eluted with ethyl acetate–methanol–water (100:13.5:10 *v/v*) and then subjected to preparative HPLC using a gradient of methanol to water with 2% formic acid (1000 mL) as eluent to obtain two pure compounds: compound 2 (18 mg) and compound **3** (7 mg). Fraction F5 (52.3 mg) was analysed by GC-MS analysis to found 4 compounds (compounds **4**, **5**, **6**, **7**).

#### 3.10.2. MALDI-TOF MS for Characterization of Condensed Tannins

The precipitated tannin sample is dissolved in methanol (10 mg/ml). The material used is 2,5 dihydroxybenzoic (DHB, 100 mg/ml in a 50/50 water/acetonitrile mixture). To improve the formation of ions, the water contains NaCl (1 mg/ml). A mixture of 10 μl of the tannin sample and 10 μl of the DHB matrix is prepared and then 1 μl of this mixture is deposited on a stainless steel plate and dried at room temperature. The recording is made with a MALDI-TOF mass spectrometry Ultraflex III (Bruker Daltonics, Bremen, Germany), equipped with a NdYAG laser, 266 nm at 200 Hz. The MALDI-TOF spectra is recorded in positive reflector mode using Na^+^ as adducts. Each spectrum is obtained after 1000 shots of laser.

#### 3.10.3. GC-MS

GC-MS analysis was performed using QP2010-Shimadzu (Shimadzu, Kyoyo, Japan) equipment operating in the EI mode at 70 eV. An SLB5 columnDB-5 ms (30 m, 0.25 mm film thickness) was employed with a 36 min temperature program of 60–320 at 10 °C/min followed by a 10 min hold at 320 °C. The injector temperature was 250 °C, the flow rate of the carrier gas (helium) was 1 mL/min, and the split ratio was 1:50. The interval of the scan *m/z* was between 35 and 900 and the identification of the compounds is based on an individual spectrum comparison of each compound in the Shimadzu NIST08 data.

#### 3.10.4. NMR

The NMR spectra (^1^H, ^13^C, COSY, HSQC, HMBC) were recorded on a Bruker Avance III 400 MHz spectrometer (Bruker biospin, Billerica, USA), Bruker BBFO Probe. The coupling constant (*J*) is expressed in Hz.

#### 3.10.5. LC-HRESIMS

Liquid Chromatography Hight Resolution-Mass chromatography (LC HRESIMS) analysis was performed using a micrOTOF-Q^TM^ (Bruker Daltonics, Bruker, Bremen, Germany) apparatus operating in the ESI positive and negative mode, set capillary on 4500v, dry gas 10 ml/min, set dry heater 190 °C.

### 3.11. Statistical Analysis

The measurements are repeated three times and the data are expressed mean + standard error of the mean (SEM). Statistical significance was defined as *p* < 0.05.

## 4. Conclusions

The results obtained suggest that *A. seyal* bark could be a potential source of active natural compounds like flavonoids, steroids, triterpenes, and tannins as catechin and gallocatechin-based oligomers. This work also evaluated the antioxidant and antibacterial activities of *A. seyal* bark extracts. The bark is rich in tannins, which could be responsible for the observed antioxidant activity but not the antibacterial activity. However, campesterol combined with stigmasterol, clionasterol and oleamide was proven to show antibacterial activity against *S. aureus*. The cytotoxicity of *A. seyal* water bark extract revealed in this study against the MRC-5 cells indicated that the safety of the bark of this plant in the traditional medicine in Djibouti should be verified by other experiments including in vivo tests and clinical studies.

## Figures and Tables

**Table 1:** Total polyphenols content (TPC) and total flavonoids content (TFC), extractable tannins of two extracts of *A. seyal* bark.

Extracts	TPC (mg GAE/100 g)	TFC (mg QE/100 g)	Extractable Tannins (%)
Methanolic extract	1927.1 ± 11.1	30.9 ± 1.5	82.8 ± 2.3%
Water extract	1820.5 ± 13.6	13.8 ± 5.3	80.7 ± 1.1%

GAE: Gallic acid Equivalent. QE: Quercetin Equivalent. Values are representative of three independent determinations. *p* values ≤ 0.05.

**Table 2:** Antioxidant activity of bark extracts of *A. seyal*.

Extracts, Fractions and Standards	DPPH IC_50_ (µg/mL)	ABTS IC_50_ (µg/mL)	FRAP (µg VE/g)
Methanolic crude extract	150 ± 2.2	27 ± 1.3	45.7 ± 6.1
Filtrate FM	250 ± 12	50 ± 3	20.7 ± 2.3
Precipitate PM	95 ± 3	16 ± 0.8	54.4 ± 9.3
Water crude extract	183 ± 5.3	33 ± 2	5.7 ± 1.0
Filtrate FW	321 ± 13	41 ± 1	3.7 ± 1.6
Precipitate PW	136 ± 5	21 ± 1	6.7 ± 1.1
Vitamin C	70 ± 7	70 ± 1.5	ND
Trolox	110 ± 2.5	18 ± 3.3	ND

Values are representative of three independent determinations. *p* values ≤ 0.01. VCE: Vitamin C Equivalent; ND: not determined; FM: filtrate devoid of tannin from methanolic extract; PM: precipitate of tannins from methanolic extract; FW: filtrate devoid of tannin from water extract; PW: precipitate of tannins from water extract.

**Table 3:** Antibacterial activity of extracts and isolated compounds from *Acacia seyal* bark expressed as MICs (µg/mL).

Extracts, Fractions and Pure Compounds	*Staphylococcus aureus*	*Pseudomonas aeruginosa*	*Corynebacterium* *urealyticum*
**Water extract**	512	>1024	>1024
**Methanolic extract**	64	512	64
**PM**	512	>1024	>1024
**FM**	32	512	32
**F1**	>256	>256	>256
**F2**	128	>256	256
**F3**	>256	>256	>256
**F4**	>256	>256	>256
**F5**	128	128	128
**F6**	256	256	>256
**F7**	128	256	>256
**F8**	128	128	>256
**F9**	256	>256	>256
**F10**	>256	>256	>256
**F11**	>256	128	256
**F12**	32	>1024	128
**Compound 1**	512	>1024	512
**Compound 2**	>1024	>1024	>1024
**Compound 3**	1024	1024	1024

**Table 4:** MALDI-TOF MS of condensed tannins from methanolic and water extracts of *Acacia seyal* bark.

Fraction	Series	[M+Na] Experimental	[M+Na] Calculated	N1	N2	N3	N2+ DHB	DP	Polymer
**Precipitate (PM) from Methanol Extract**									
PM	S1	737.3	737.1	0	2	0	1	**2**	Dimer
PM	S1	1025.3	1025.2	0	3	0	1	**3**	Trimer
PM	S1	1329.4	1329.3	1	3	0	1	**4**	Tetramer
PM	S1	1633.4	1633.3	2	3	0	1	**5**	Pentamer
PM	S2	889.3	889.2	0	3	0	0	**3**	Trimer
PM	S2	905.2	905.2	1	2	0	0	**3**	Trimer
PM	S2	921.2	921.2	2	1	0	0	**3**	Trimer
PM	S2	1193.0	1193.3	1	3	0	0	**4**	Tetramer
PM	S2	1481.4	1481.3	1	4	0	0	**5**	Pentamer
PM	S2	1769.5	1769.4	1	5	0	0	**6**	Hexamer
PM	S2	2057.5	2057.4	1	6	0	0	**7**	Heptamer
PM	S2	2345.6	2345.6	1	7	0	0	**8**	Octamer
**Precipitate (PW) from Water Extract**									
PW	S3	617.2	617.1	1	1	0	0	**2**	Dimer
PW	S3	905.2	905.2	1	2	0	0	**3**	Trimer
PW	S3	1193.3	1193.3	1	3	0	0	**4**	Tetramer
PW	S3	1481.4	1481.3	1	4	0	0	**5**	Pentamer
PW	S3	1769.4	1769.4	1	5	0	0	**6**	Hexamer
PW	S3	2057.5	2057.4	1	6	0	0	**7**	Heptamer
PW	S3	2345.6	2345.5	1	7	0	0	**8**	Octamer
PW	S3	2633.6	2633.6	1	8	0	0	**9**	Nonamer
PW	S4	769.2	769.1	1	1	1	0	**2**	Dimer
PW	S4	1057.2	1057.2	1	2	1	0	**3**	Trimer
PW	S4	1345.3	1345.3	1	3	1	0	**4**	Tetramer
PW	S4	1633.4	1633.3	1	4	1	0	**5**	Pentamer

N1—Gallocatechin monomer with molecular weight 304.0583, N2—Catechine monomer with molecular weight 288.0634, N3—Gallic acid monomer with molecular weight 152.010956, DHB—(2,5dihydroxybenzoic) matrix with molecular weight 136.016, S1–S2—precipitate of tannins from water extract (PW), S3–S4—precipitate of tannins from methanol extract (PM), DP—Degree of polymerization.

## Supplementary Materials

The following are available online at https://www.mdpi.com/1420-3049/25/10/2392/s1. In the supplementary materials it is possible to find: in Figure S1 the picture of the *Acacia seyal* plant; in Figure S2 the linear correlation between ABTS and DPPH of IC_50_ of methanol and water *Acacia seyal* extracts; in Figure S3–S4 EIMS and ^1^H NMR spectra of lupeol; in Figure S5–S10 HRESIMS, ^1^H, ^13^C, COSY, HSQC, HMBC of Epicatechin; in Figure S11–S17 HRESIMS, ^1^H, ^13^C, COSY, HSQC, HMBC of Catechin; in Figure S18–S21 EIMS spectra of Clionasterol, Stigmasterol, Campesterol and Oleamide respectively.

## Abbreviations

VC	Vitamin C
QE	Quercetin Equivalent
VCE	Vitamin C Equivalent
GAE	Gallic acid Equivalent
TPC	Total polyphenols content
TFC	Total flavonoids content
FM	filtrate devoid of tannin from methanolic extract
PM	precipitate of tannins from methanolic extract
FW	filtrate devoid of tannin from water extract
PW	precipitate of tannins from water extract
