# Supplementary material for: Evaluation of Antioxidant and Antibacterial Activities, Cytotoxicity of Acacia seyal Del Bark Extracts and Isolated Compounds
Source: Molecules. 2020 May 21;25(10):2392. doi: 10.3390/molecules25102392 (PMC7288156; doi:10.3390/molecules25102392)

SUPPLEMENTARY MATERIALS

Evaluation of antioxidant and antibacterial activities, cytotoxicity of *Acacia seyal* Del bark extracts and isolated compounds.

Abdirahman Elmi[1,2], Rosella Spina[1], Arnaud Risler[1], Stéphanie Philippot[1], Ali Mérito[2], Raphaël E. Duval[1,3], Fatouma Mohamed Abdoul-latif [2], Dominique Laurain-Mattar[1]*

[1] Université de Lorraine, CNRS, L2CM, F-54000 Nancy, France; rosella.spina@univ-lorraine.fr (R.S.), arnaud.risler@univ-lorraine.fr (A.R.), stephanie.philippot@univ-lorraine.fr (S.P.), raphael.duval@univ-lorraine.fr (R.E.D.),
[2] Medicinal Research Institute, Centre d’Etudes et de Recherche de Djibouti, IRM-CERD, Route de l’Aéroport, Djibouti; abelfourreh@hotmail.com (A.E.), fatouma_abdoulatif@yahoo.fr (F.M.A), alimerito@hotmail.fr (A.M.)
[3] ABC Platform®, Faculté de Pharmacie, F-54505 Vandoeuvre-lès-Nancy, France

***** Correspondence: dominique.mattar@univ-lorraine.fr (D.L.M.)

Contents

Figure S1: *Acacia seyal*, localité de Day, région de Tadjourah (Nord de Djibouti), altitude 1313 m.
Figure S2: Linear correlation between ABTS and DPPH of IC_{50} of methanol and water *Acacia seyal* extracts.
Figure S3: EIMS of Lupeol (compound 1).
Figure S4: ^{1}H NMR of Lupeol (compound 1, 400 MHz, $CDCl_3$)
Figure S5: HRESIMS of Epicatechin (compound 2).
Figure S6: ^{1}H NMR of Epicatechin (compound 2, 400 MHz, CD_3OD)
Figure S7: ^{13}C NMR of Epicatechin (compound 2, 100 MHz, CD_3OD).
Figure S8: ^{1}H-^{1}H COSY spectrum of Epicatechin (compound 2).
Figure S9: HSQC spectrum of Epicatechin (compound 2)
Figure S10: HMBC spectrum of Epicatechin (compound 2).
Figure S11: HRESIMS of Catechin (compound 3).
Figure S12: ^{1}H NMR of Catechin (compound 3, 400 MHz, CD_3OD)
Figure S13: ^{13}C NMR of Catechin (compound 3, 100 MHz, CD_3OD)
Figure S14: ^{1}H-^{1}H COSY spectrum of Catechin (compound 3).
Figure S15: Enlarged ^{1}H-^{1}H COSY spectrum of Catechin (compound 3).
Figure S16: HSQC spectrum of Catechin (compound 3).
Figure S17: HMBC spectrum of Catechin (compound 3).
Figure S18: EIMS of Clionasterol (compound 4).
Figure S19: EIMS of Stigmasterol (compound 5)
Figure S20: EIMS of Campesterol (compound 6).
Figure S21: EIMS of Oleamide (compound 7).

Figure S1: *Acacia seyal*, localité de Day, région de Tadjourah (Nord de Djibouti), altitude 1313 m.

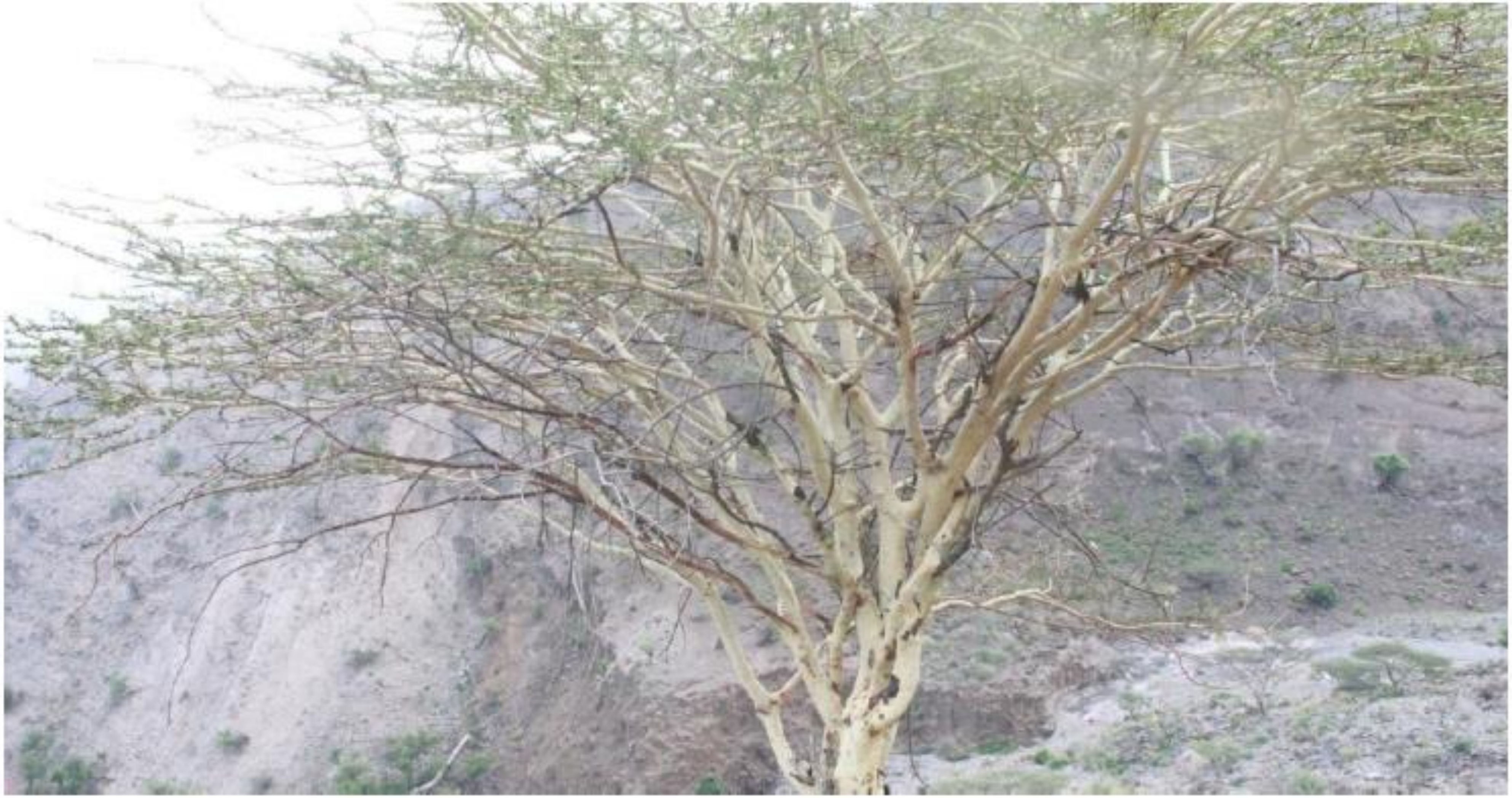

Figure S2: Linear correlation between ABTS and DPPH of IC_{50} of methanol and water *Acacia seyal* extracts.

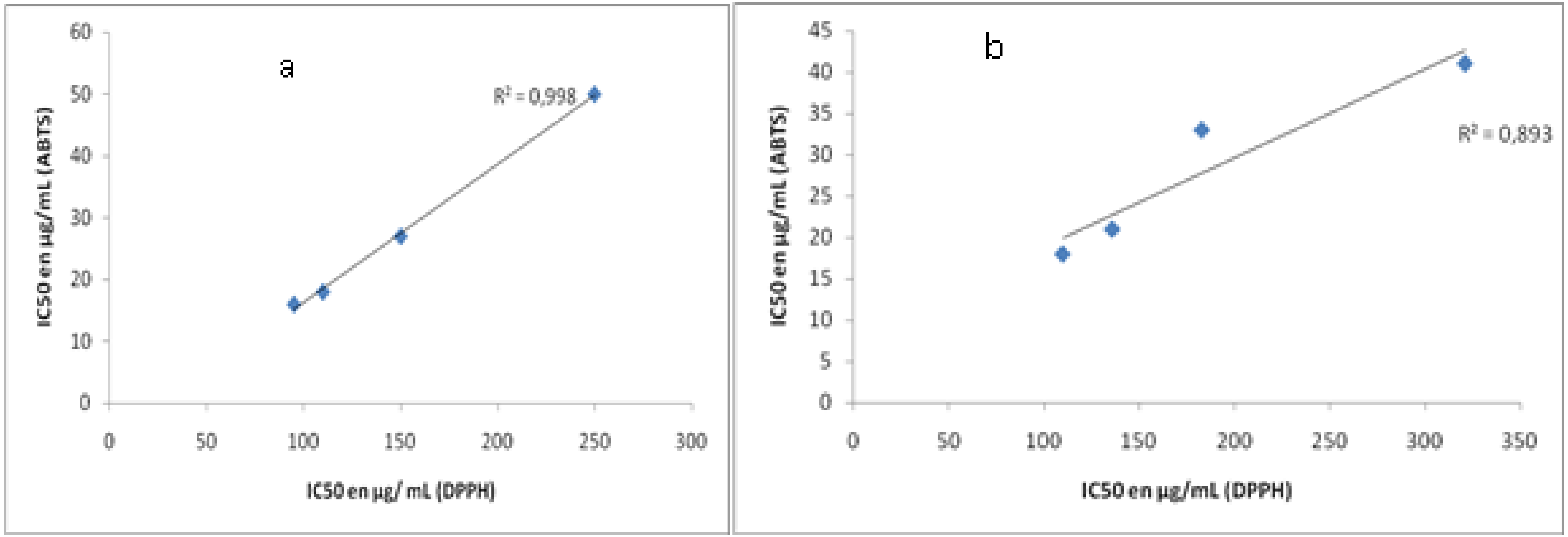

Figure S3: EIMS of Lupeol (compound 1).

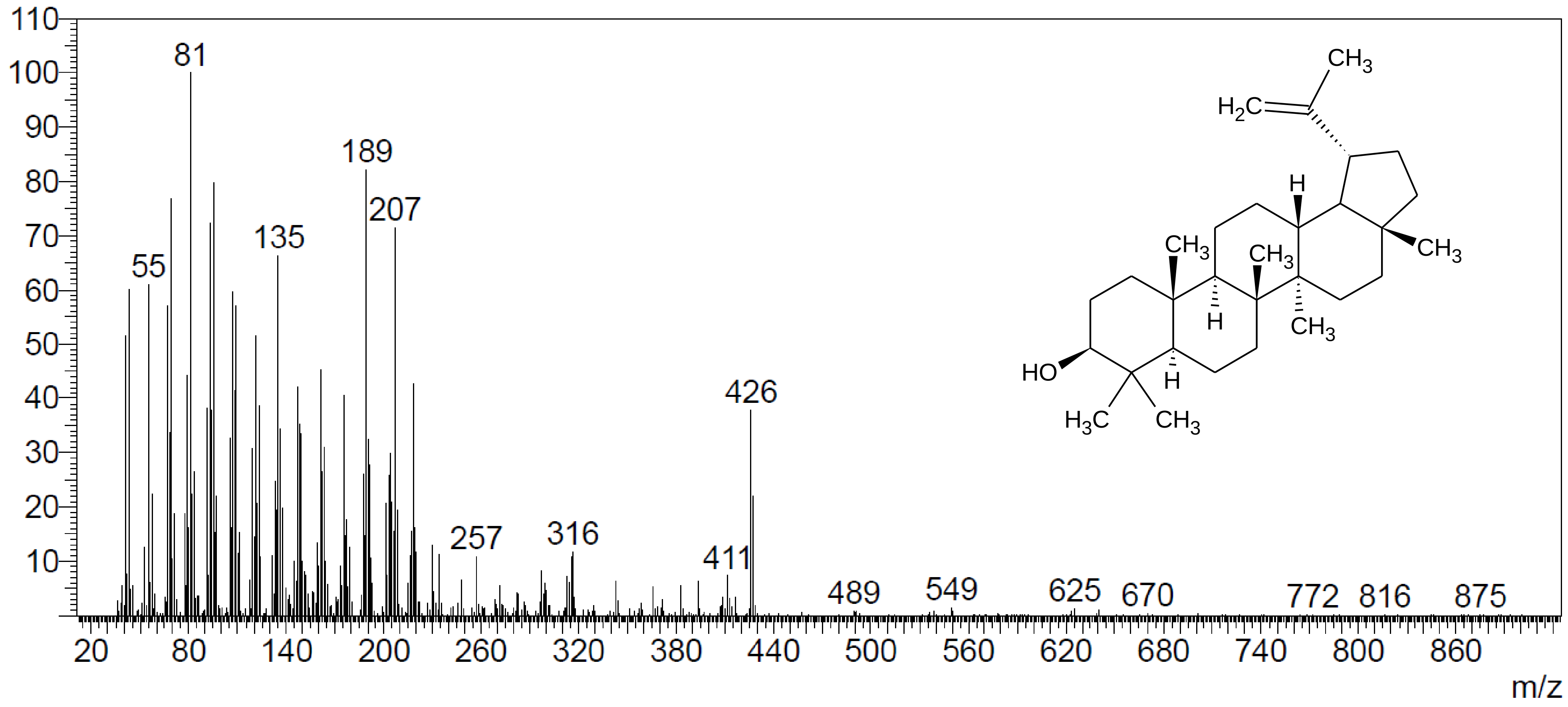

Figure S4: ^{1}H NMR of Lupeol (compound 1, 400 MHz, $CDCl_3$)

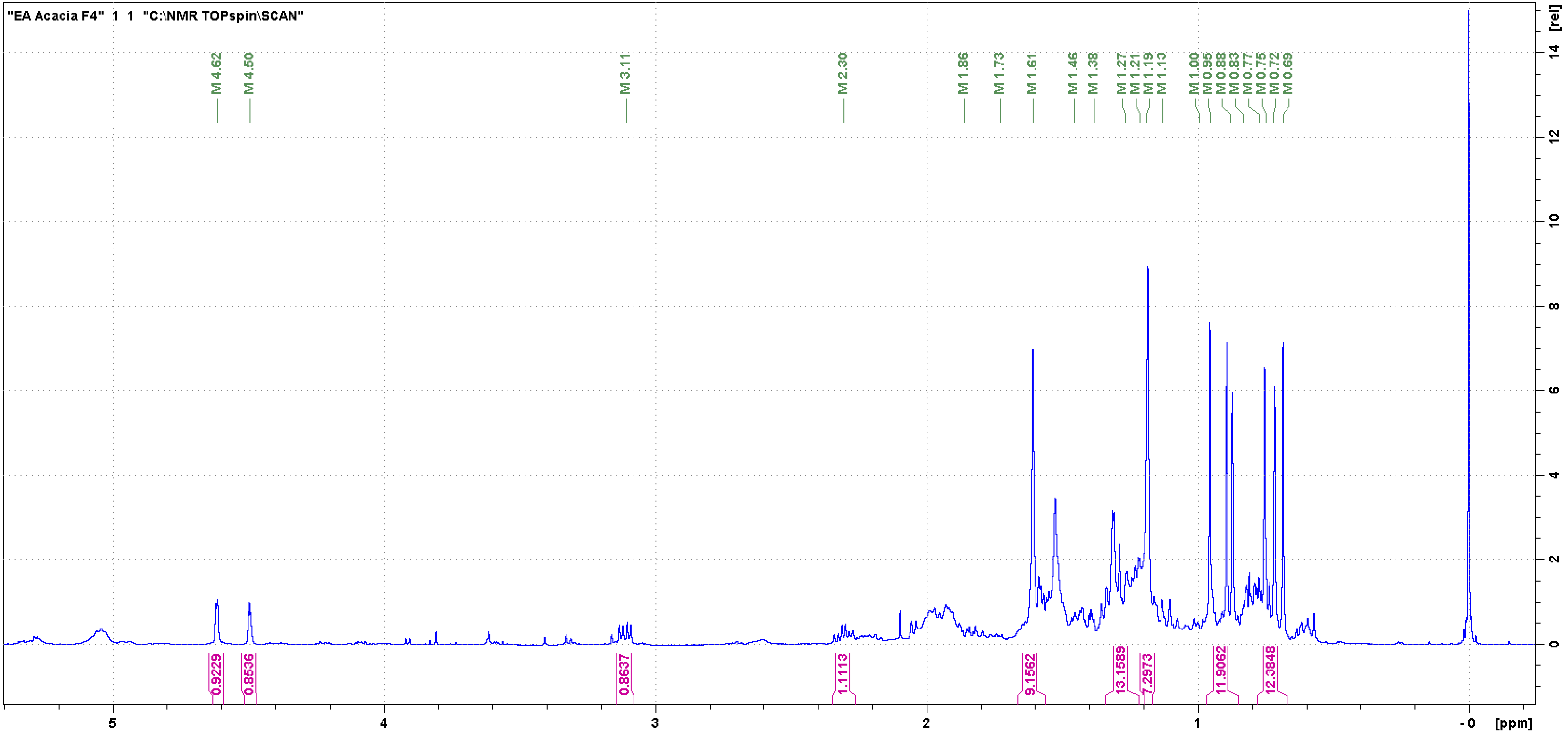

Figure S5: HRESIMS of Epicatechin (compound 2).

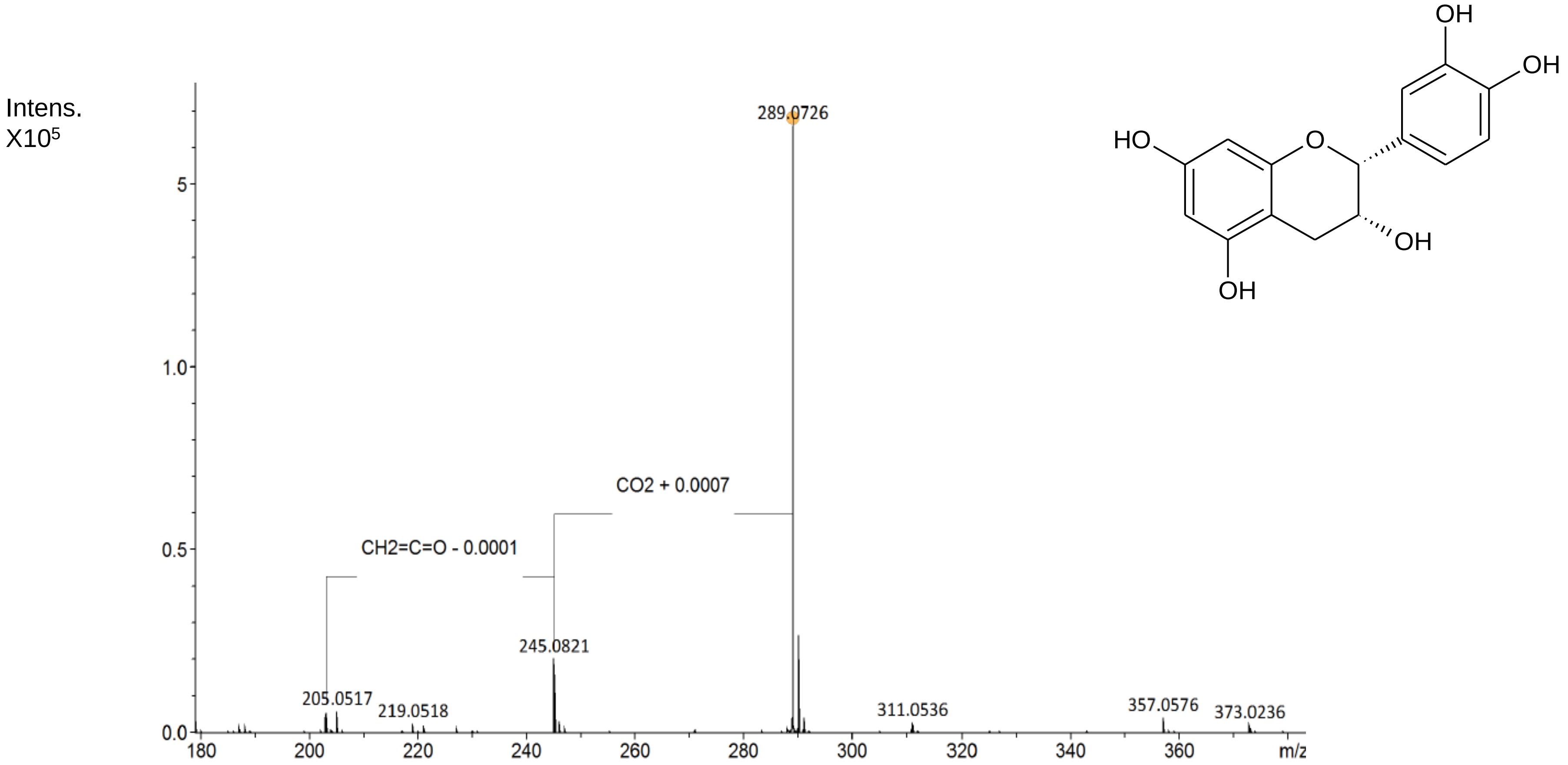

Figure S6: ^{1}H NMR of Epicatechin (compound 2, 400 MHz, CD_3OD)

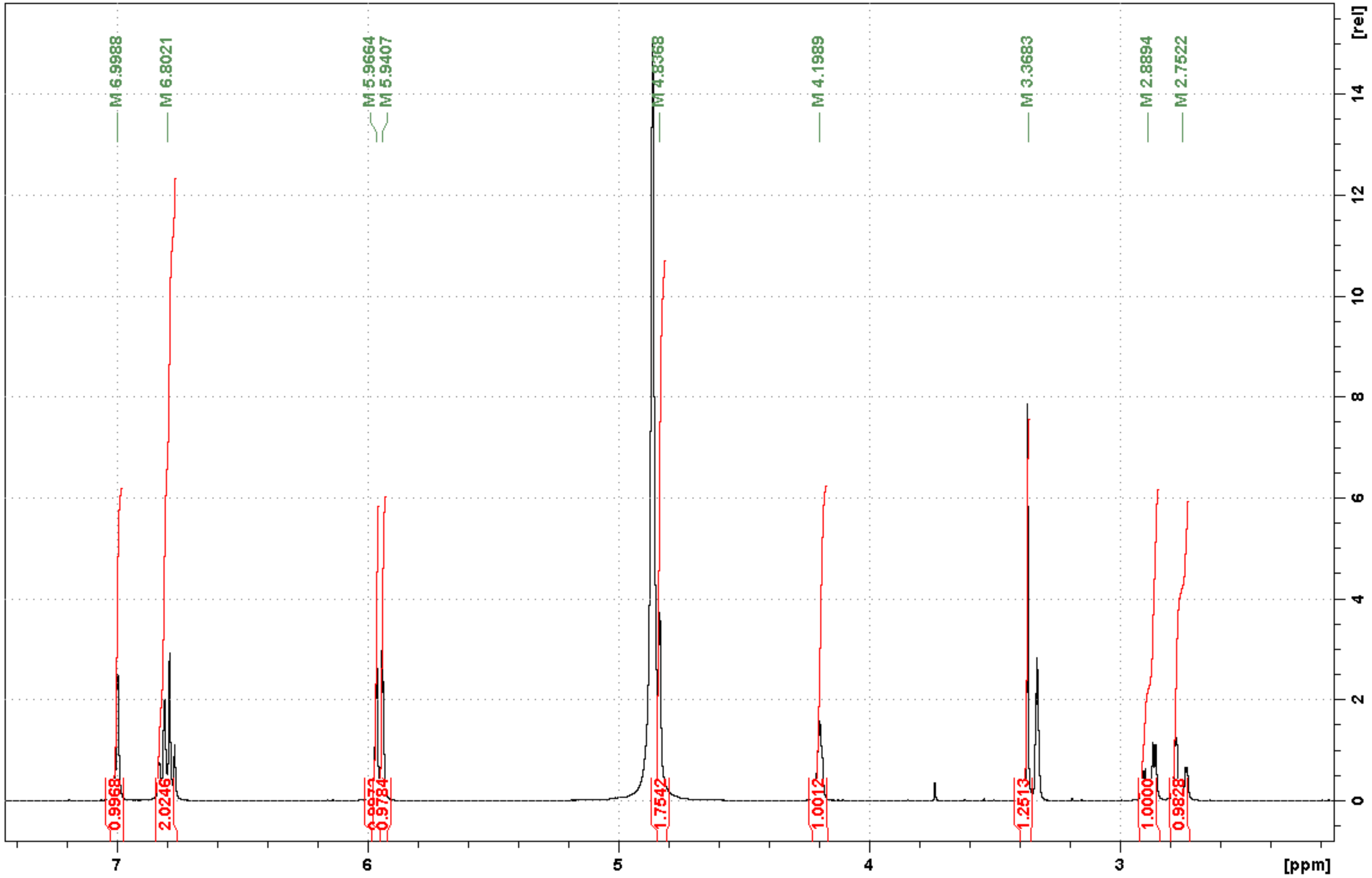

Figure S7: ^{13}C NMR of Epicatechin (compound 2, 100 MHz, CD_3OD).

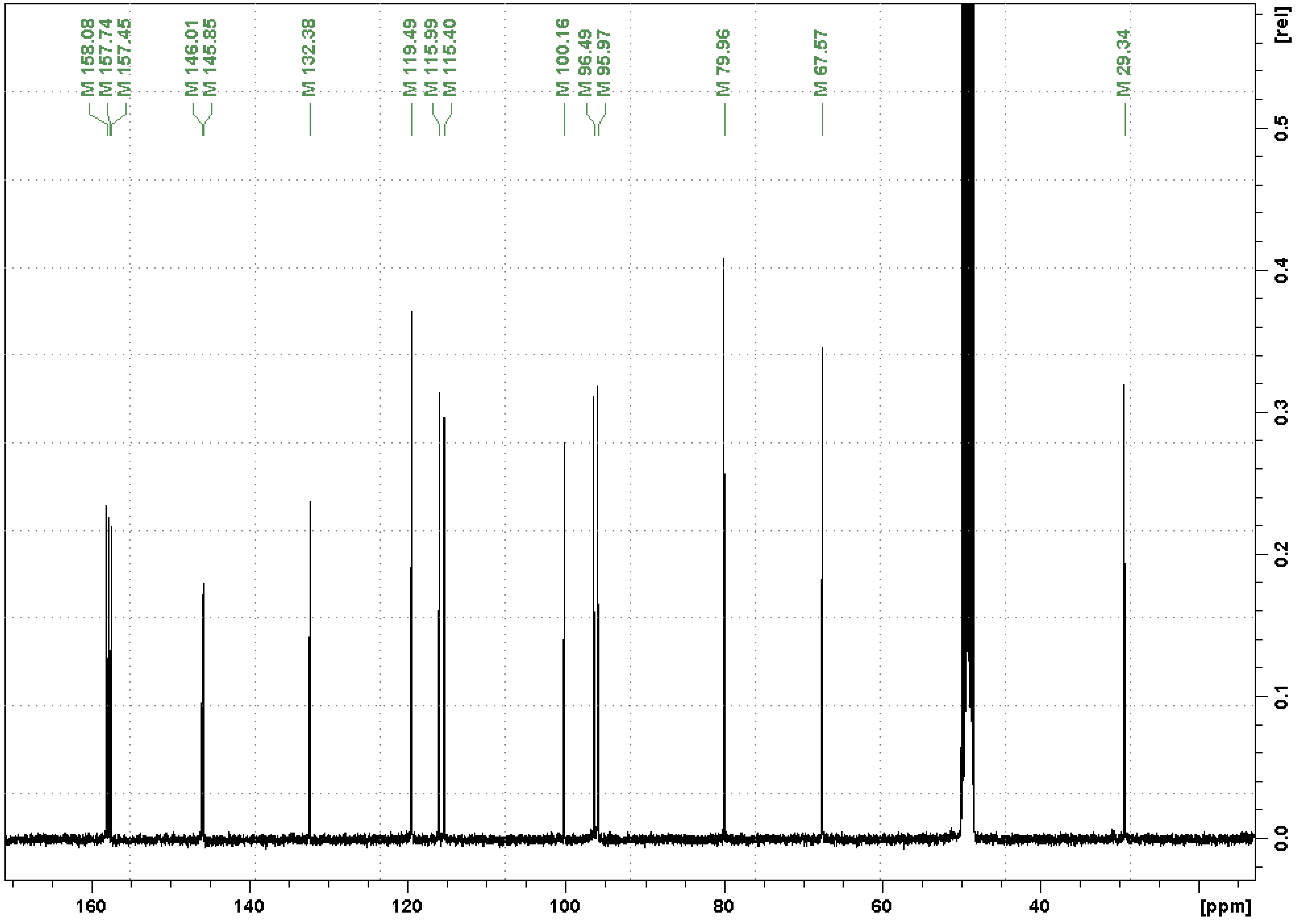

Figure S8: ^{1}H-^{1}H COSY spectrum of Epicatechin (compound 2).

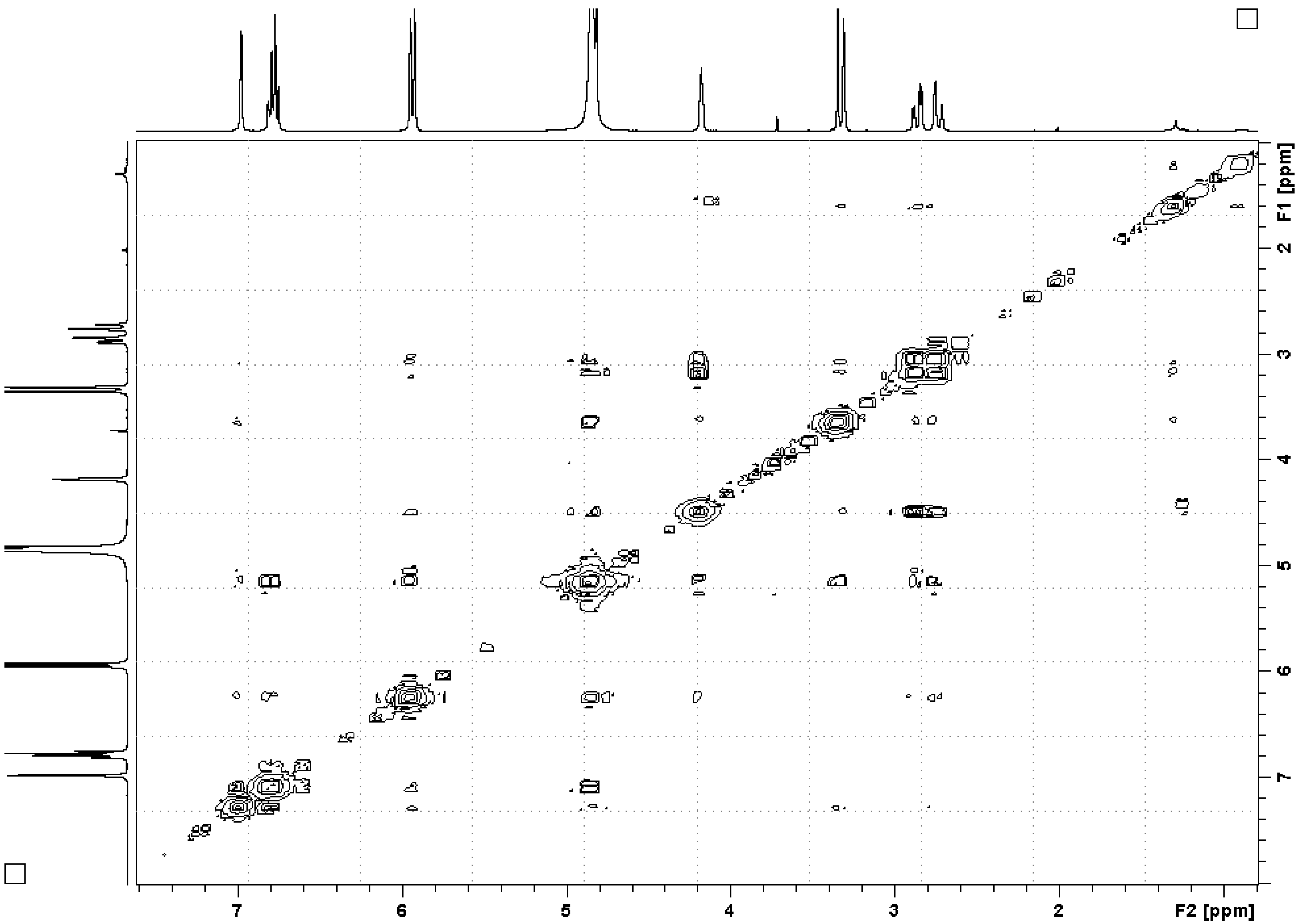

Figure S9: HSQC spectrum of Epicatechin (compound 2)

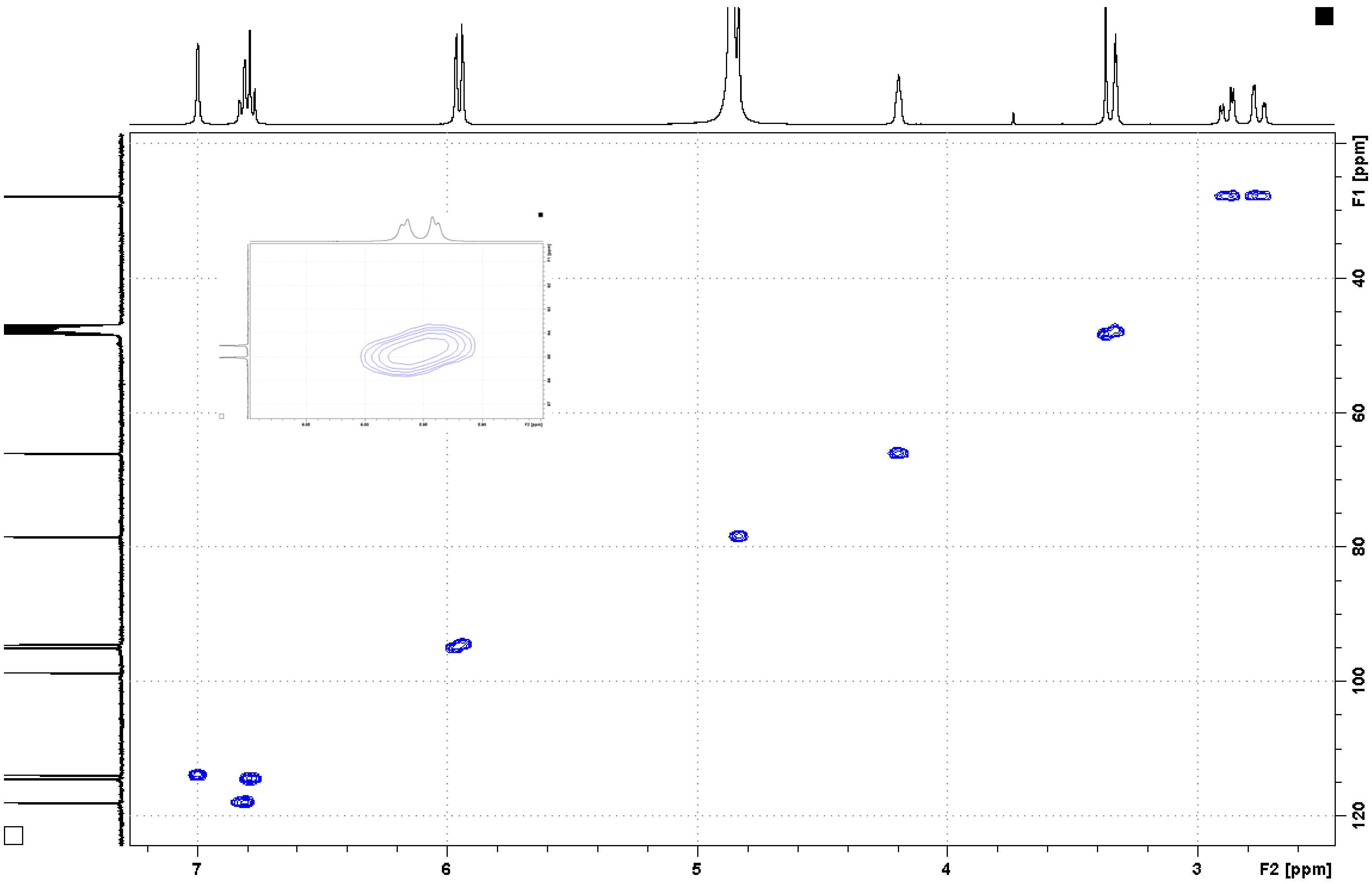

Figure S10: HMBC spectrum of Epicatechin (compound 2).

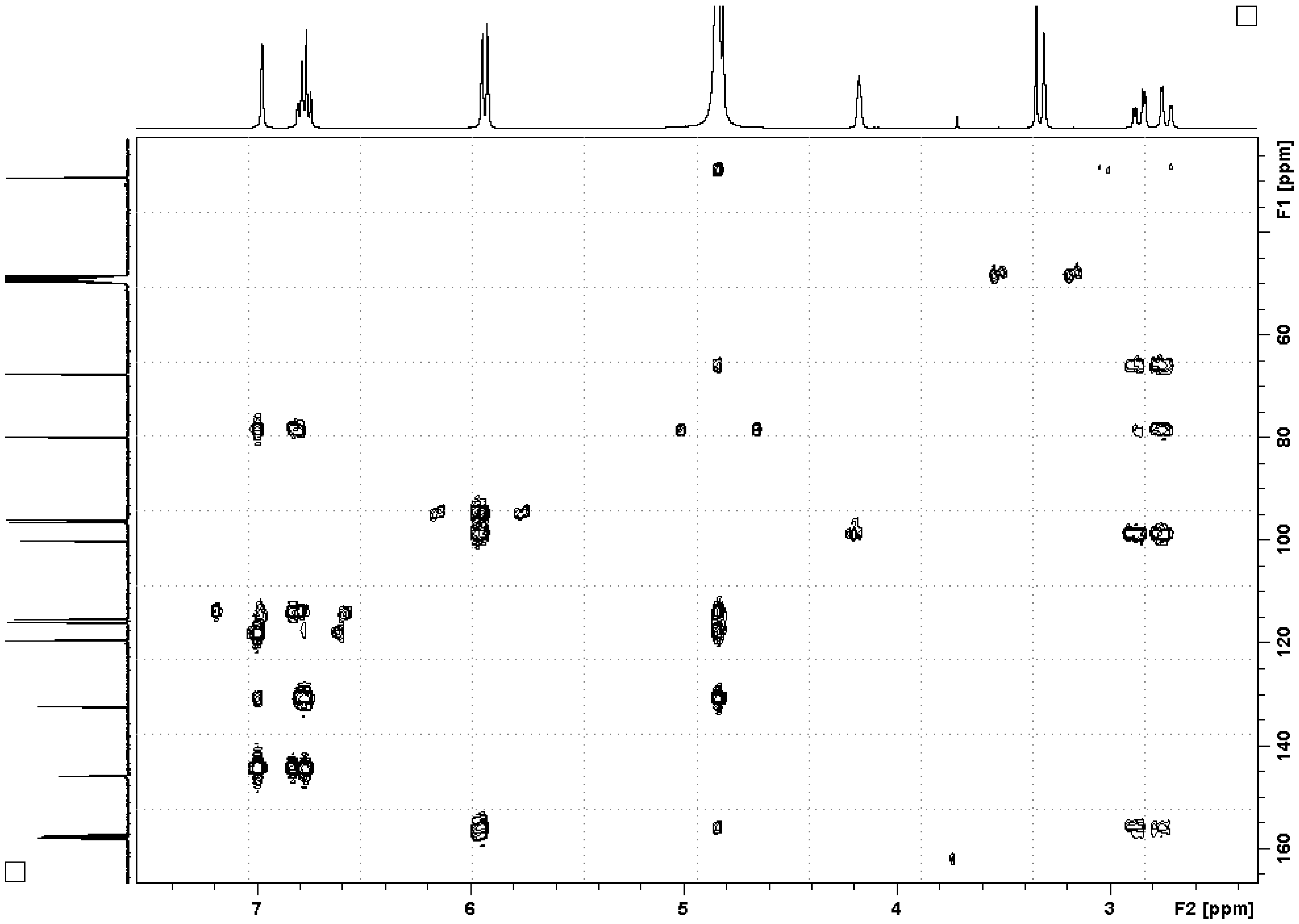

Figure S11: HRESIMS of Catechin (compound 3).

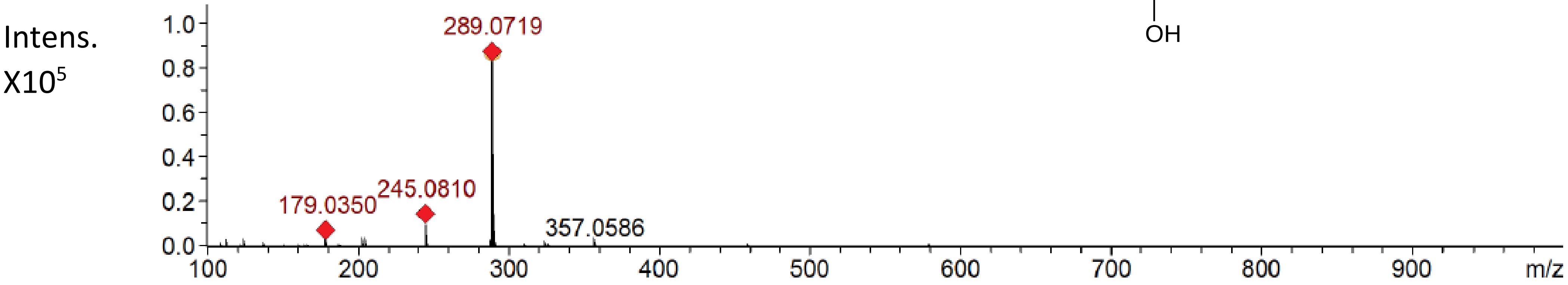

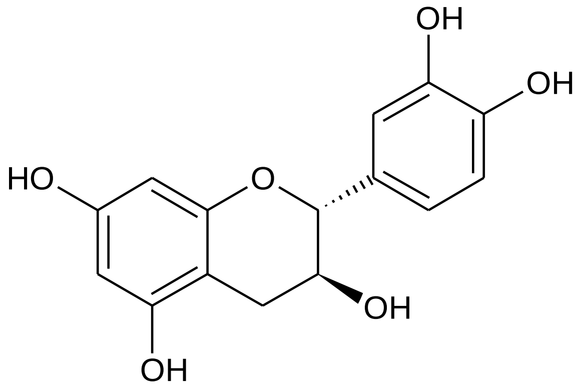

Figure S12: ^{1}H NMR of Catechin (compound 3, 400 MHz, CD_3OD)

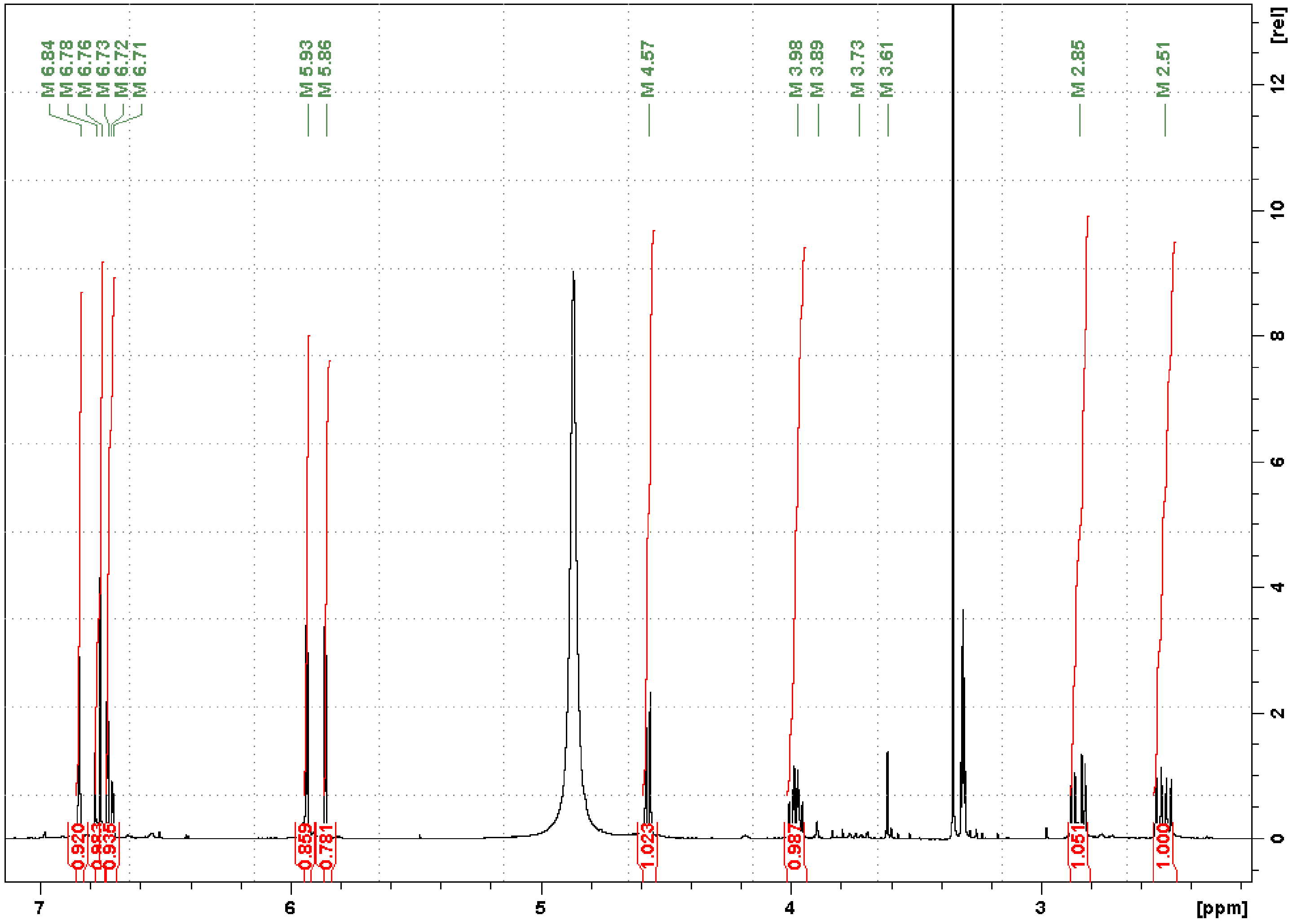

Figure S13: ^{13}C NMR of Catechin (compound 3, 100 MHz, CD_3OD)

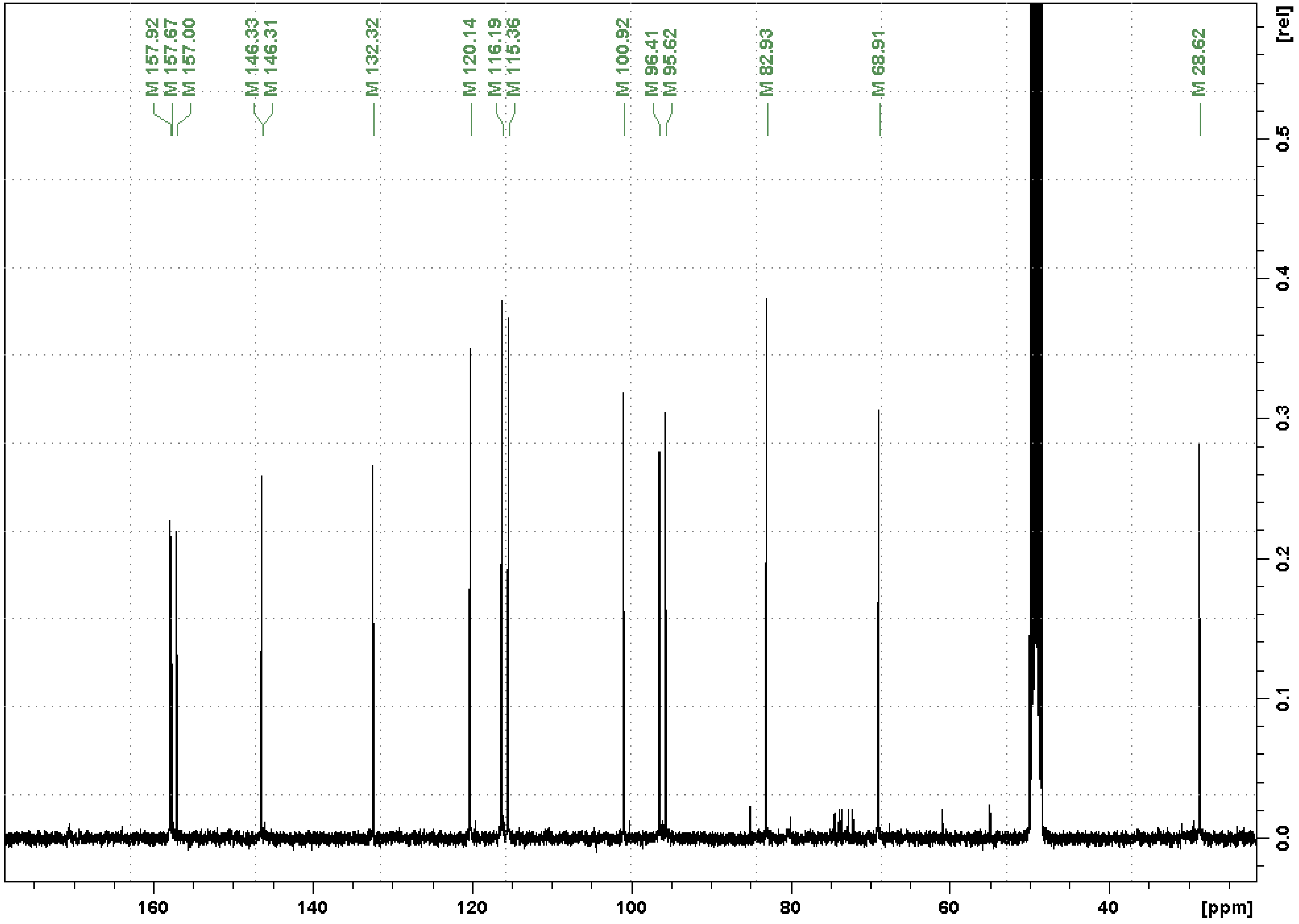

Figure S14: ^{1}H-^{1}H COSY spectrum of Catechin (compound 3).

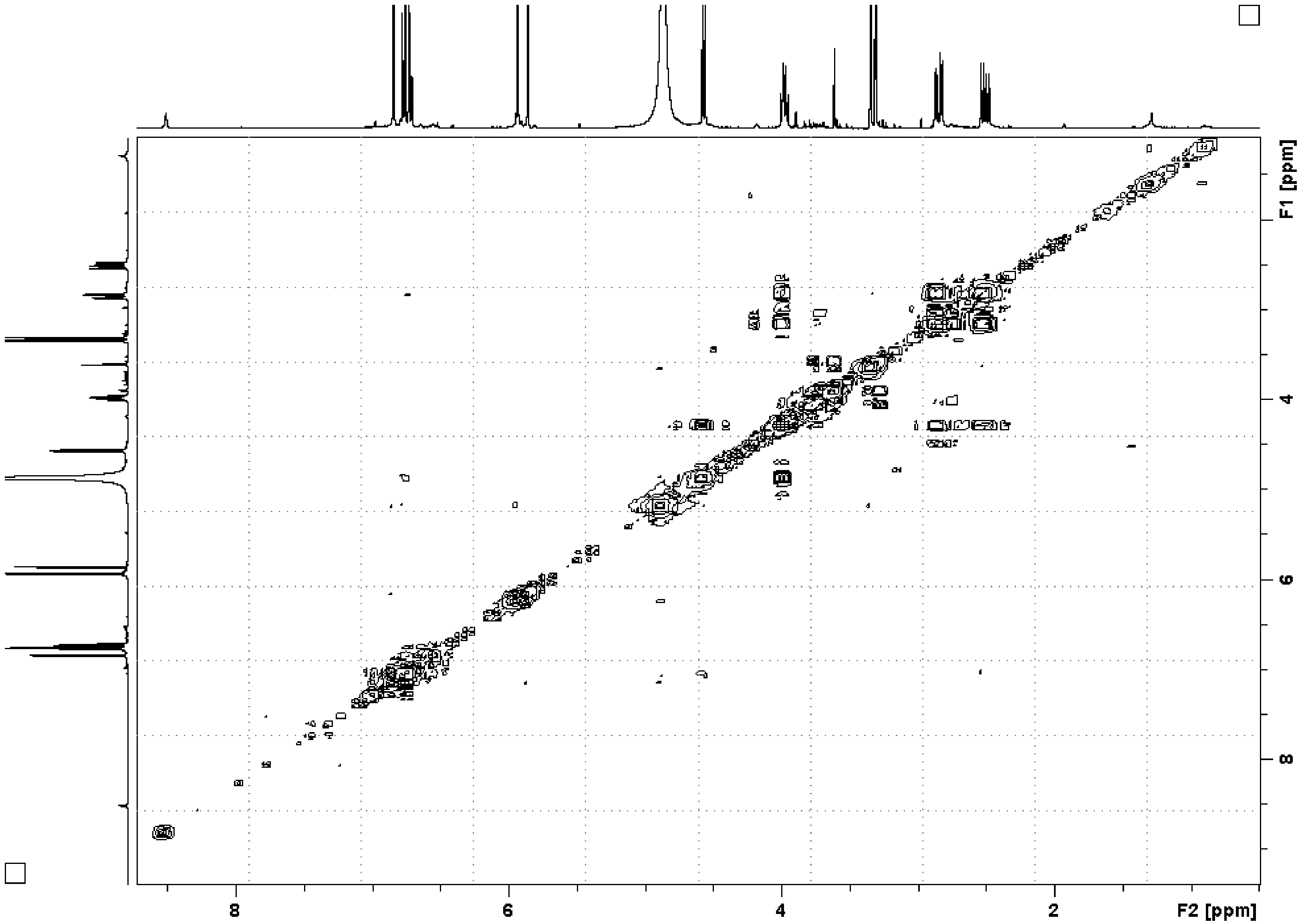

Figure S15: Enlarged ^{1}H-^{1}H COSY spectrum of Catechin (compound 3).

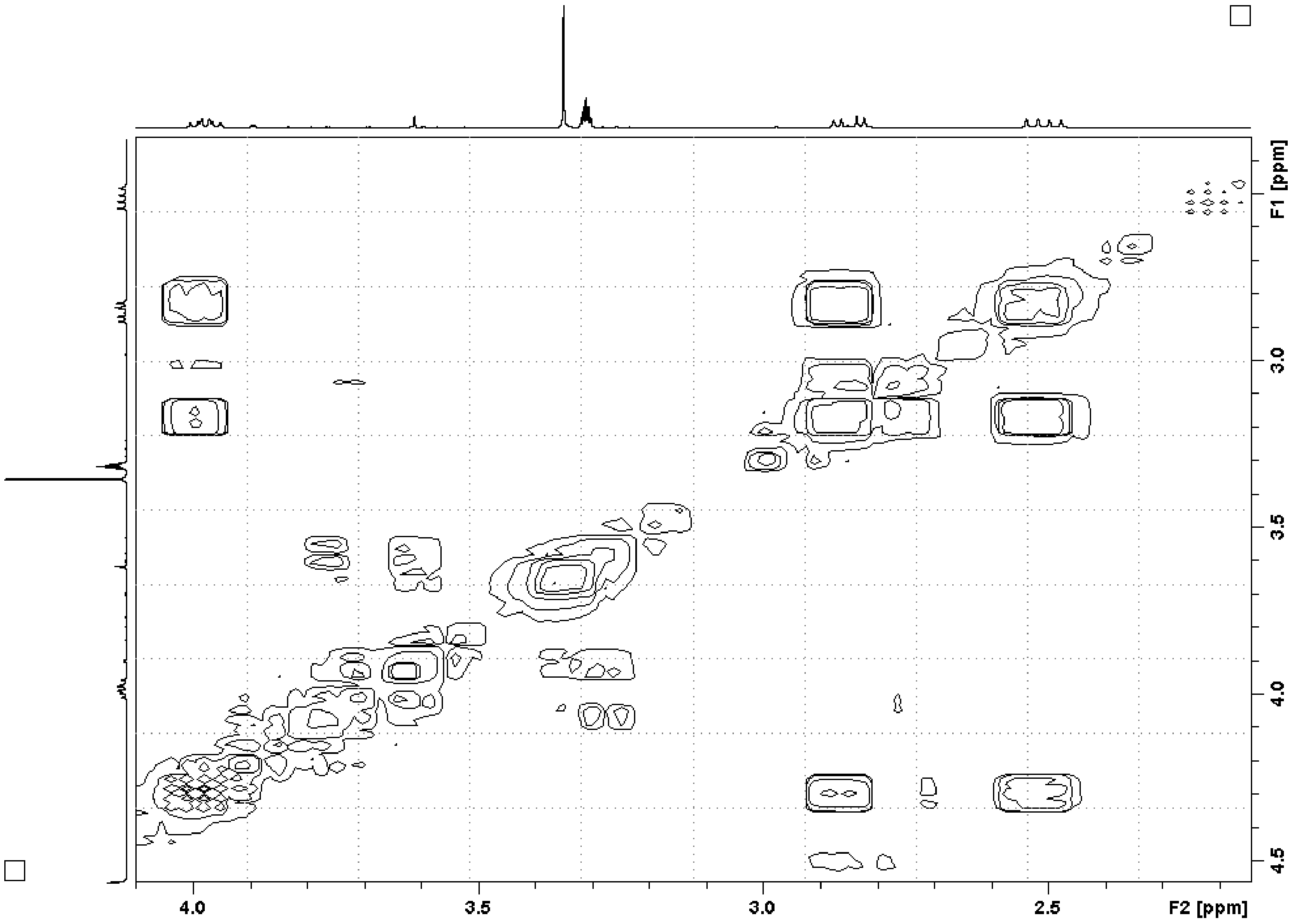

Figure S16: HSQC spectrum of Catechin (compound 3).

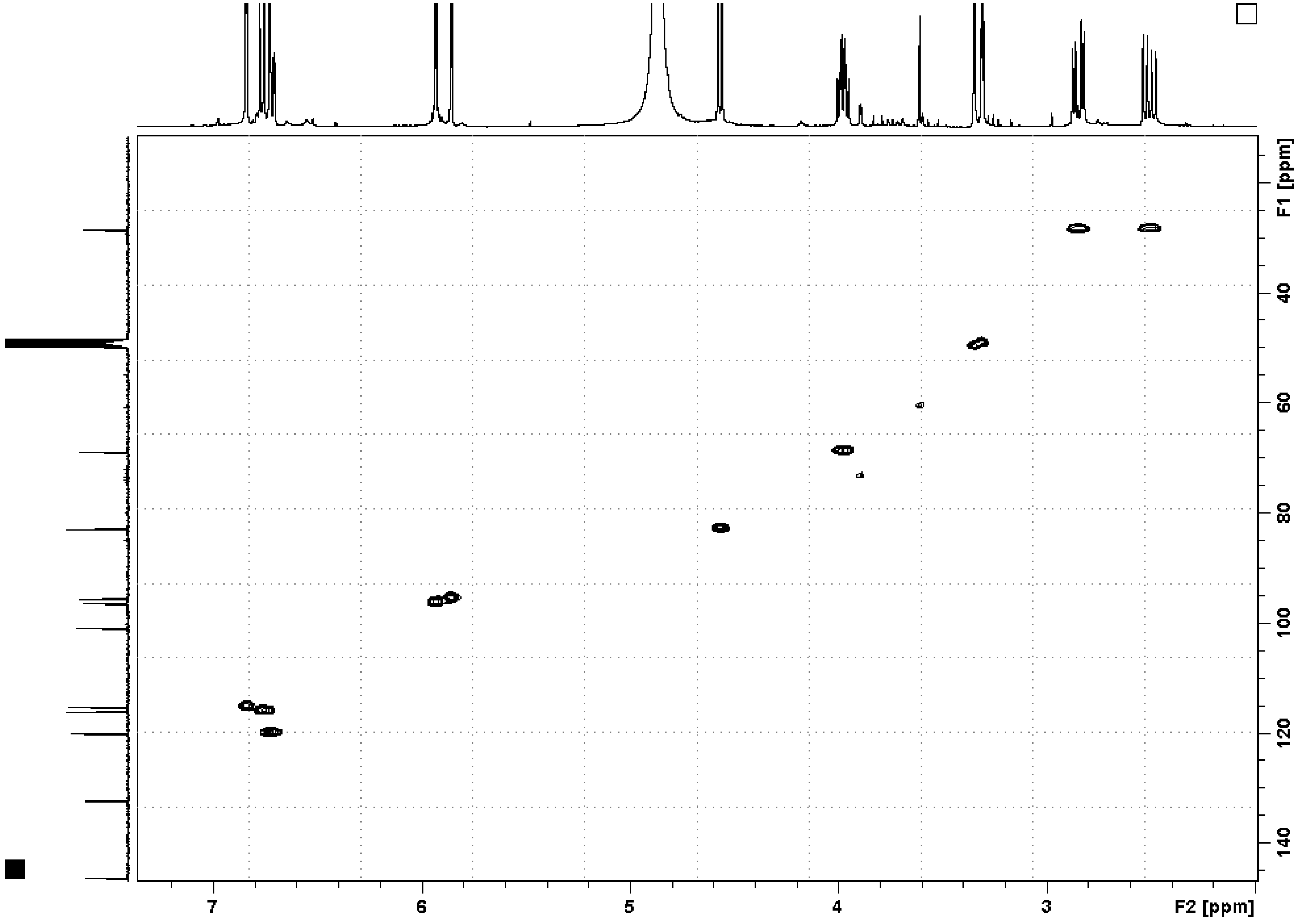

Figure S17: HMBC spectrum of Catechin (compound 3).

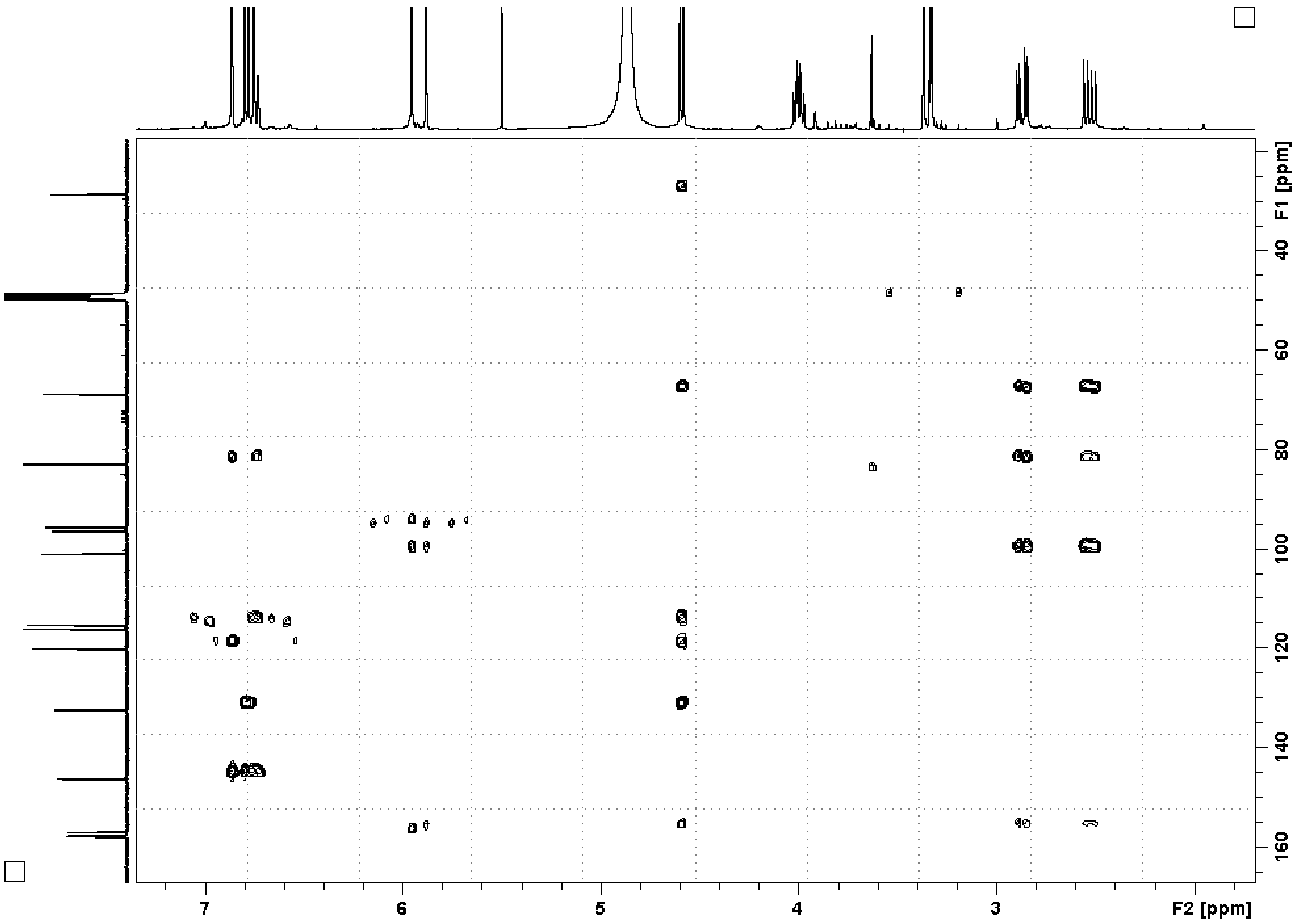

Figure S18: EIMS of Clionasterol (compound 4).

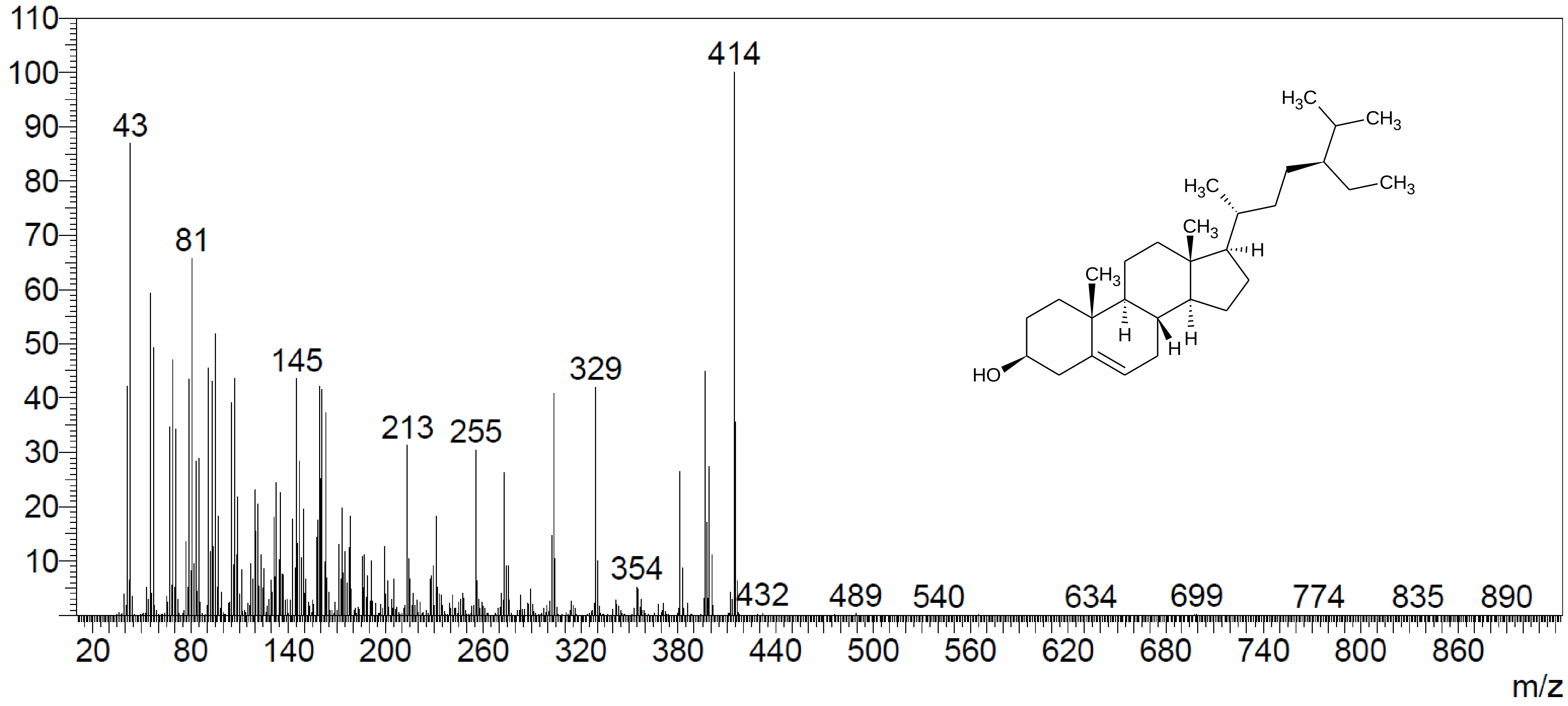

Figure S19: EIMS of Stigmasterol (compound 5).

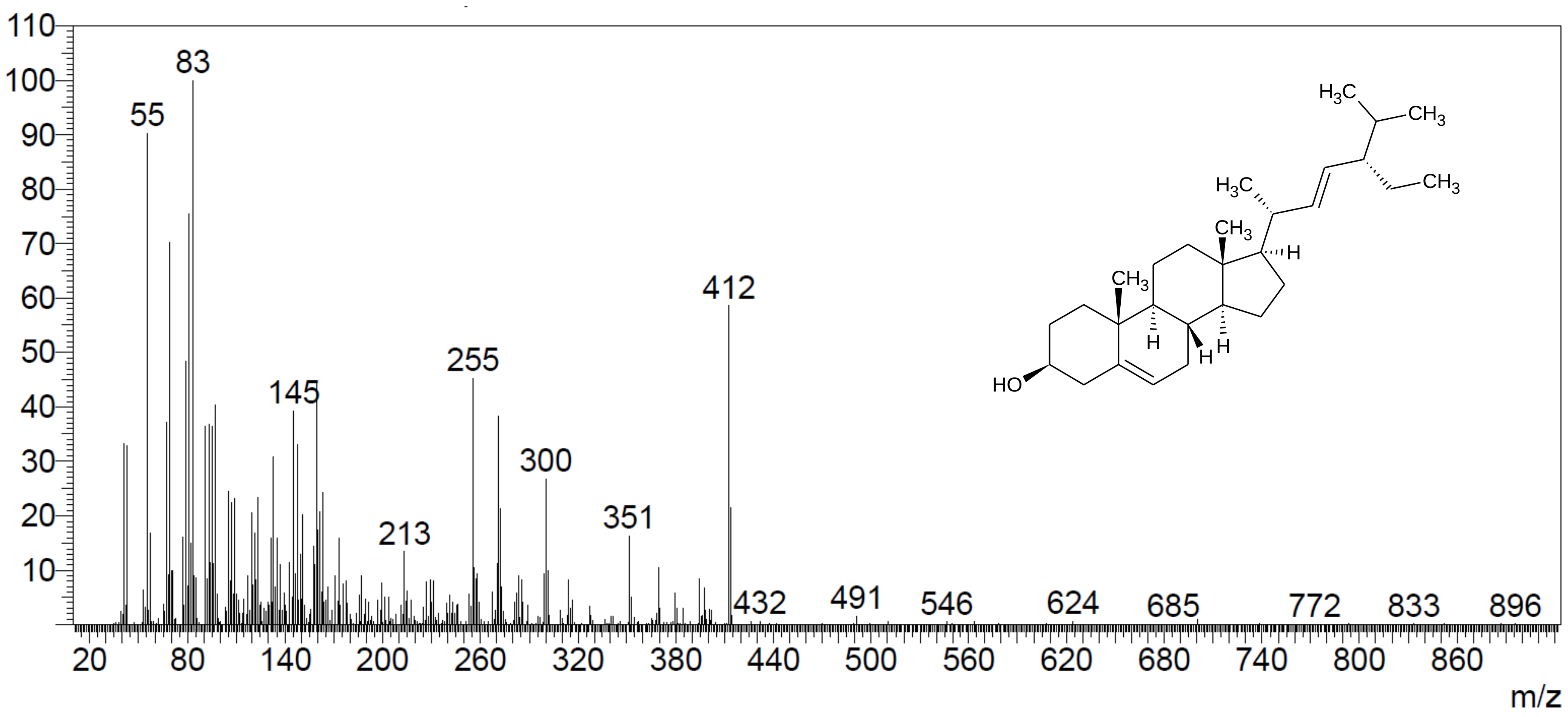

Figure S20: EIMS of Campesterol (compound 6).

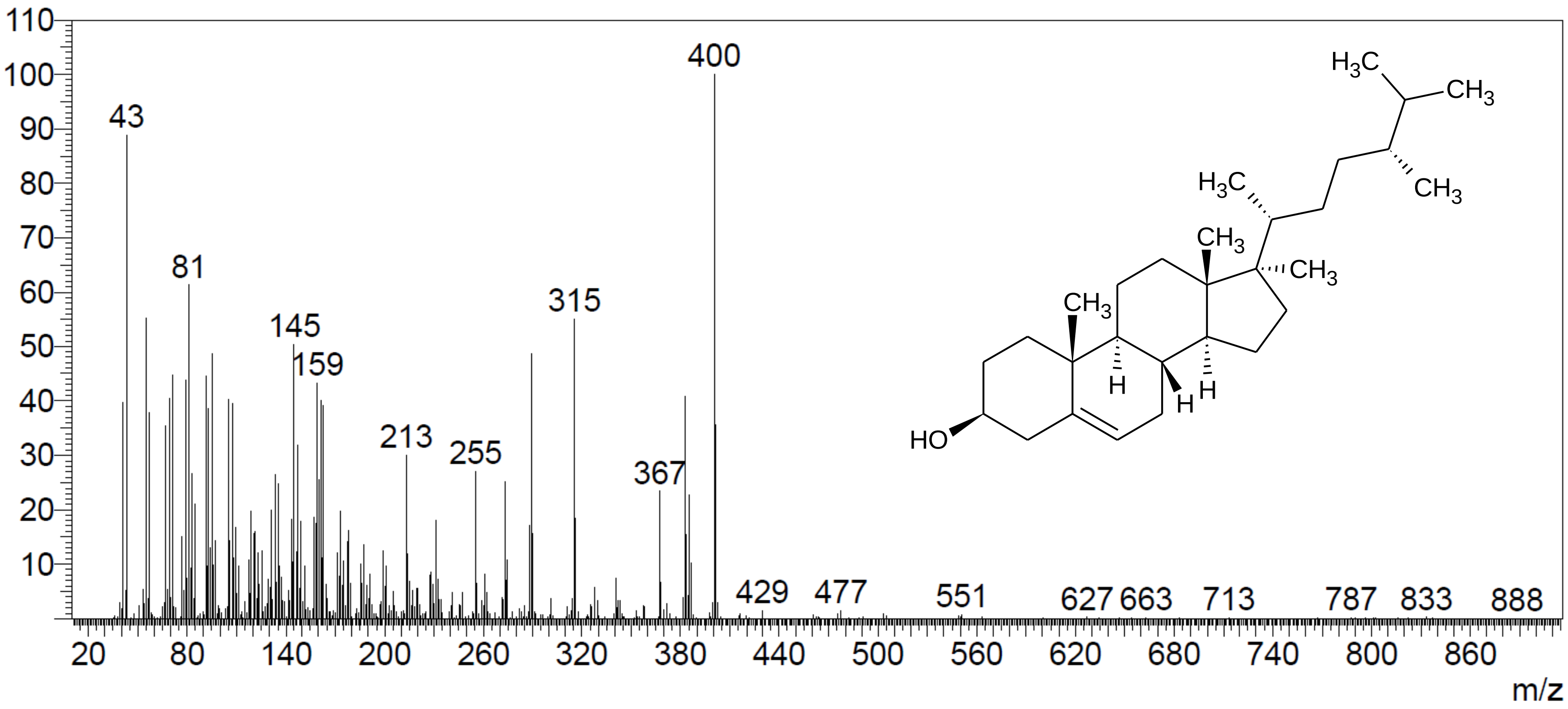

Figure S21: EIMS of Oleamide (compound 7).

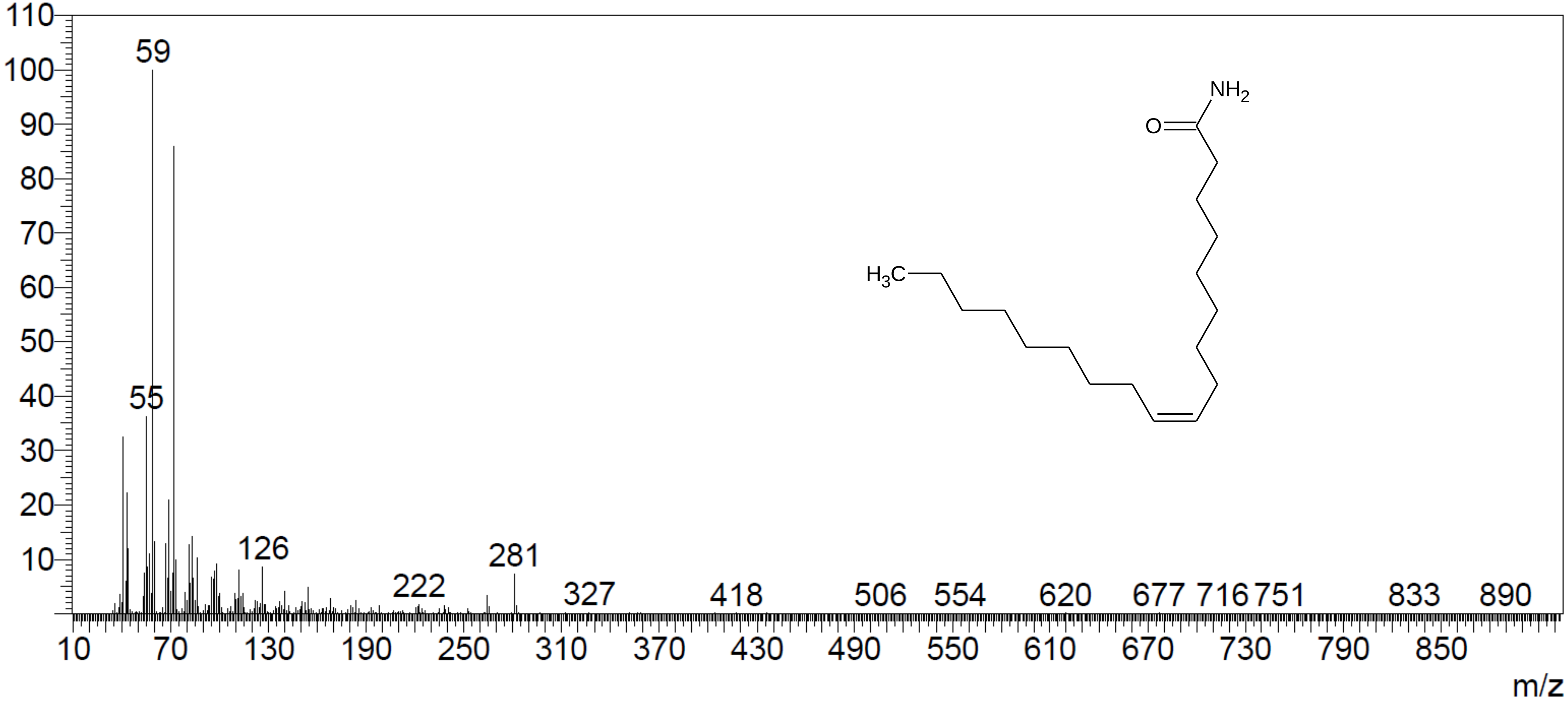

Supplement: Supplementary file 1 [file molecules-25-02392-s001.pdf]
